# Pregnancy and obstetric-neonatal outcomes of patients with thin endometrium using three different endometrial preparation protocols in frozen embryo transfer cycles: a historical cohort of 2671 patients

**DOI:** 10.1186/s12978-025-02166-z

**Published:** 2025-10-15

**Authors:** Liu Jiang, Haoming Huang, Jiayin Zhou, Yan Li, Yueping Zhou, Kun Qian

**Affiliations:** Reproductive Medicine Center, Tongji Hospital, Tongji Medicine College, Huazhong University of Science and Technology, No. 1095, Jiefang Avenue, Qiaokou District, Wuhan, 430030 People’s Republic of China

**Keywords:** Endometrial preparation, Frozen embryo transfer, Thin endometrium, Clinical pregnancy, Obstetric-neonatal outcome

## Abstract

**Background:**

Endometrial thickness independently predicts pregnancy outcomes in frozen embryo transfer (FET) cycles. Thin endometrium always results in implantation failure and worse obstetric-neonatal outcomes. However, it has not been reported which endometrial preparation strategy achieved optimal outcomes in patients with thin endometrium undergoing FET cycles.

**Methods:**

This historical cohort study was conducted on 2671 women with thin endometrium who underwent their first FET cycle at the Reproductive Medicine Center of a university-affiliated hospital between January 2018 and August 2022 (followed up to August 2023). Patients were divided into three groups according to endometrial preparation protocols (NC: natural cycle, AC: artificial cycle, GnRH-a + AC: AC with gonadotropin-releasing hormone agonist pretreatment). Thin endometrium was defined as endometrial thickness < 8 mm on the first day of progesterone administration. Patients with uterine abnormalities, recurrent spontaneous abortion, or donor oocytes were excluded. We also further analyzed the condition of endometrial thickness < 7 mm. Pregnancy and obstetric-neonatal outcomes were assessed.

**Results:**

A total of 2671 patients were included in the study. Among patients with endometrial thickness < 8 mm, the clinical pregnancy rate was 36.2% (691/1908) in the AC group, 35.2% (178/506) in the GnRH-a + AC group, and 33.9% (87/257) in the NC group. The live birth rates were 26.8% (512/1908), 25.3% (128/506), and 27.6% (71/257) in the three groups, respectively. No statistical differences were observed in pregnancy rates or obstetric-neonatal outcomes in pairwise comparisons, except that the biochemical pregnancy loss rate in the NC group was significantly lower than that in the AC group (3.9% versus 8.6%, *P* < 0.05). Furthermore, this result remained consistent after multivariate logistic regression (crude odds ratio [95% CI]: 0.428 [0.223,0.821], adjusted odds ratio [95% CI]: 0.444 [0.230,0.856]). For patients with endometrial thickness < 7 mm, there were no significant differences in any outcomes across the three groups.

**Conclusions:**

Analysis using the 8 mm cut-off revealed a lower biochemical pregnancy loss rate in the NC group compared to the AC group. In contrast, no significant differences were observed in clinical pregnancy, live birth, or obstetric-neonatal outcomes based on endometrial preparation strategy for patients with an endometrial thickness < 7 mm or 8 mm.

**Supplementary Information:**

The online version contains supplementary material available at 10.1186/s12978-025-02166-z.

## Introduction

 In vitro fertilization (IVF) is the mainstay of contemporary infertility therapy. More than 2 million treatment cycles are carried out globally every year [1–3], with a notable increase in frozen embryo transfer (FET) cycles attributed to the advancements in vitrification techniques [4–7]. However, studies show that FET is linked to higher rates of hypertensive disorders of pregnancy (HDP) and preeclampsia compared to fresh embryo transfers. This increased risk may be due to differences in the number of corpora lutea present during the cycle [8–10]. Therefore, it is important to explore ways to improve pregnancy and obstetric-neonatal outcomes after FET. One key approach is investigating different endometrial preparation protocols used before embryo transfer.

The various protocols for endometrial preparation remain a topic of debate within the general population [11–15] and in specific groups, such as those with polycystic ovary syndrome (PCOS) [16–18] or endometriosis [19, 20]. A key aspect of this debate is the trade-off between convenience and safety. The popularity of the artificial cycle (AC) protocol may be due to its convenience in scheduling embryo transfer while achieving similar pregnancy outcomes to the natural cycle (NC). Accumulating evidence reported that artificial cycles may be associated with increased maternal risks, particularly HDP and preeclampsia [21, 22]. In addition, recent studies suggest that the use of natural cycles was associated with reduced risks of miscarriage, as well as increased rates of clinical pregnancy and live birth [23–25]. Evaluating endometrial preparation protocols is crucial for patients with thin endometrium. As insufficient thickness leads to poorer fresh transfer outcomes [26], a freeze-all strategy is often adopted [27]. Consequently, FET is often used for individuals with thin endometrium, a known independent predictor of pregnancy outcomes that frequently causes implantation failure [28–31]. In addition to reduced pregnancy rates and increased miscarriage risks, it is also associated with higher rates of obstetric-neonatal complications [32–35]. Guidelines therefore recommend evaluating the impact of endometrial preparation protocols on pregnancy outcomes in these patients [36]. However, relevant literature remains limited, with no reports on obstetric-neonatal complications. Since numerous studies in general populations have shown that NC decreases the risk of these complications compared to AC [11, 12], it is worth investigating whether this benefit extends to patients with thin endometrium. Some studies indicated the positive effectiveness of stimulated protocols in pregnancy outcomes with a thin endometrium [37–39], not to mention the comparison of NC and AC. While the AC with downregulation of gonadotropin-releasing hormone agonist (GnRH-a) improved the clinical outcomes of embryo transfer [40], its impact on FET with thin endometrium is unclear.

The present study aimed to assess the impact of various endometrial preparation strategies on outcomes in women with thin endometrium. We conducted a historical cohort study to evaluate the pregnancy and obstetric-neonatal outcomes among the NC, AC, and GnRH-a + AC groups of patients with thin endometrium.

## Materials and methods

### Study population

**Fig. 1:**
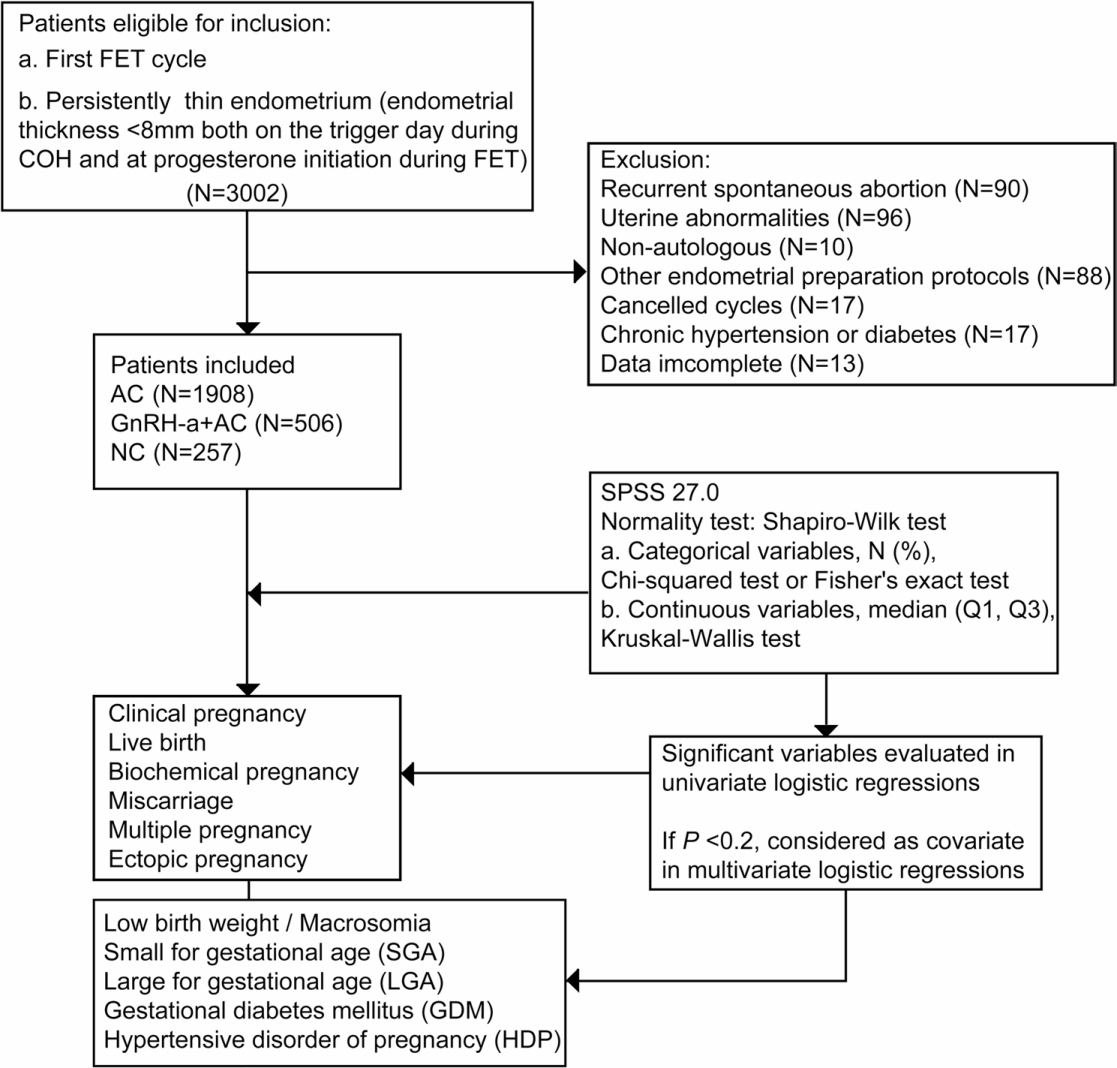
Flowchart of this study. Notes: Inclusion and exclusion criteria, statistical analysis methods, and outcome indicators. FET = frozen embryo transfer; PGT = pre-implantation genetic testing; AC = artificial cycle; NC = natural cycle; GnRH-a = gonadotropin-releasing hormone agonist

This retrospective cohort study was conducted among women with a thin endometrium (defined as an endometrial thickness < 8 mm on the first day of progesterone administration) who underwent their first FET at the Reproductive Medicine Center of a university hospital between January 2018 and August 2022. Follow-up for all patients continued until August 2023. Exclusion criteria included a history of recurrent spontaneous abortion, the presence of uterine abnormalities (such as uterine malformations, severe adenomyosis, untreated submucosal fibroids, endometrial polyps, or uterine effusion), oocyte donation, other endometrial preparation protocols, canceled cycles, chronic hypertension or diabetes, endometrial thickness ≥ 8 mm on the trigger day during controlled ovarian hyperstimulation, and incomplete data (e.g., missing values for endometrial thickness, number of embryo transfers, or type of embryo transfers). A flowchart of this study is presented in Fig. 1.

The study protocol was approved by the Declaration of Helsinki by the Institutional Review Board (IRB) of Tongji Hospital, affiliated with Tongji Medical College, Huazhong University of Science and Technology (approval number TJ-IRB20230921). Patients’ informed consent was waived.

### Endometrial preparation and embryo transfer

**Fig. 2:**
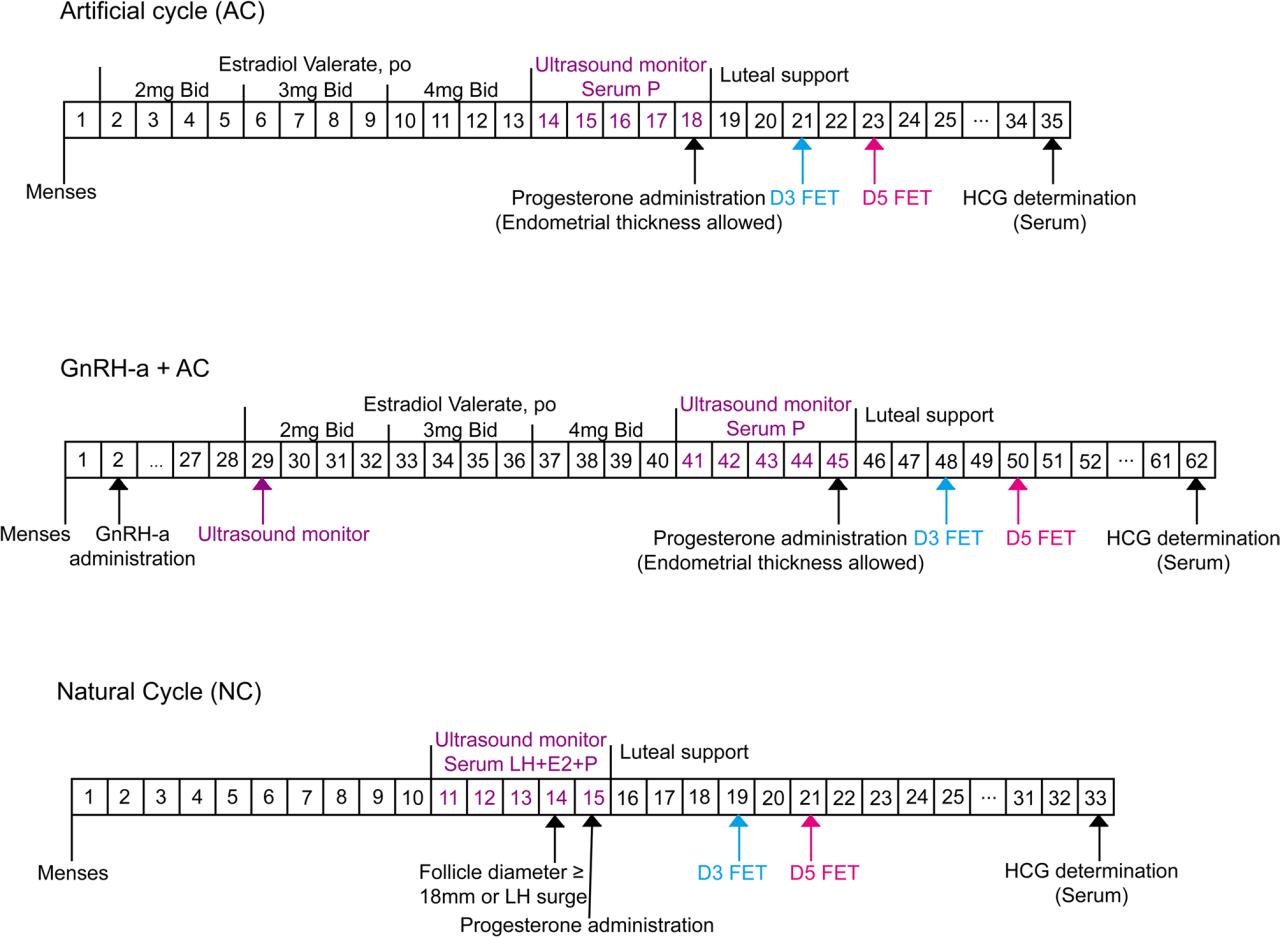
Timeline of the three endometrial preparation protocols. Notes: In the AC group, estradiol valerate was administrated, endometrial thickness was monitored, and estradiol dosage increased gradually. In the GnRH-a + AC group, patients received a single 3.75 mg injection of a GnRH agonist on day 2 of the menstrual cycle. In the NC group, endometrial thickness, follicular development, and serum hormone measurements were monitored starting on days 10–12 of the menstrual cycle. P = progesterone; AC = artificial cycle; NC = natural cycle; GnRH-a = gonadotropin-releasing hormone agonist; D3 = cleavage stage embryo; D5/6 = blastocyst; HCG = human chorionic gonadotropin

The study compared three common endometrial preparation protocols for frozen embryo transfer: the AC group, the AC group with GnRH agonist pretreatment (GnRH-a + AC), and the NC group [41, 42]. These protocols are clearly illustrated in Fig. 2. In the AC group, estradiol (Progynova, Bayer Schering Pharma AG, Germany) was administered orally, starting with 4 mg/day from day 2 to 5, increasing to 6 mg/day from day 6 to 9, and then to 8 mg/day from day 10 to 13. Endometrial thickness and serum progesterone levels were monitored on day 14 of the menstrual cycle. Once the endometrial thickness reached the desired level and serum progesterone was below 1.2 ng/ml, the embryo was transferred following daily progesterone administration for three or five consecutive days. Luteal phase support consisted of 20 mg of orally administered progesterone (Abbott Biologicals B.V., Netherlands) in conjunction with a 90 mg vaginal gel (Merck Serono Limited, Germany) until 8 to 10 weeks of pregnancy. In the GnRH-a + AC group, patients received a single 3.75 mg injection of a GnRH agonist (triptorelin or leuprorelin) on day 2 of their menstrual cycle. After a 28-day suppression period, they commenced the same AC protocol as described, followed by embryo transfer. The NC group monitored endometrial thickness, follicular development, and serum hormone levels from day 10 to 12 of the menstrual cycle until the dominant follicle measured 18 mm or detected an LH surge. Ovulation may occur naturally in the true natural cycle or be induced by the injection of human chorionic gonadotropin (HCG; Ovitrelle 250 µg, Merck, Rahway, NJ, USA) in the modified natural cycle. Additionally, all patients were routinely administered low-dose aspirin (Bayer, 100 mg/day) orally, beginning at the onset of each endometrial preparation protocol.

Embryos were vitrified using standard protocols and subsequently stored in liquid nitrogen. On the transfer day, the embryos were thawed and placed in a recovery medium at 37℃ for 2 to 3 h before assessing their viability. Only those embryos or blastocysts deemed viable were selected for transfer. The embryo transfer procedure was conducted under ultrasound guidance using a soft catheter, irrespective of the endometrial preparation protocol. Typically, a single high-quality blastocyst was transferred into the uterine cavity, positioned approximately 1.5 to 2 cm from the uterine fundus to enhance implantation potential. In our center, the strategy of elective single embryo transfer was advocated for most patients to mitigate complications associated with multiple pregnancies. However, in cases of thin endometrium, where clinical pregnancy rates are lower and miscarriage rates are higher compared to non-thin endometrium cases, no more than two embryos or blastocysts were transferred.

### Outcomes

This study evaluated both pregnancy and obstetric-neonatal outcomes. Data on pregnancy outcomes were collected from medical records, including clinical pregnancy rate (CPR, confirmed by the presence of a gestational sac on ultrasound), biochemical pregnancy loss rate (determined by a positive serum β-hCG test, but did not result in a clinical pregnancy), live birth rate (LBR, defined as the birth of a live neonate beyond 24 weeks gestation), multiple pregnancy rate (the presence of two or more gestational sacs), and the ectopic pregnancy rate (where the embryo implanted outside the uterus, diagnosed via ultrasound or clinical evaluation). The primary outcome was the LBR. To minimize bias from vanishing twin pregnancies, the analysis included only live births from singleton pregnancies for obstetric-neonatal outcomes. These outcomes included low birth weight (LBW, birth weight < 2, 500 g), macrosomia (birth weight > 4, 000 g), small-for-gestational-age (SGA, birth weight below the 10th percentile for gestational age), large-for-gestational-age (LGA, birth weight above the 90th percentile), preterm birth (birth before 37 completed weeks of gestation), gestational diabetes mellitus (diagnosed according to the criteria of the International Association of Diabetes and Pregnancy Study Groups), hypertensive disorders of pregnancy (HDP, which includes pregnancy-induced hypertension and preeclampsia), placenta previa (placenta implanted over or near the inner opening of the cervix, diagnosed by ultrasound) and fetal malformation (any structural or functional abnormality identified at birth).

### Statistical analysis

All statistical analyses were performed using SPSS 27.0. Categorical variables were compared pairwise among different endometrial preparation protocols (AC, GnRH-a + AC, and NC) using the Chi-squared test or Fisher’s exact test, as appropriate. Continuous variables were assessed for normality using the Shapiro-Wilk test. As none of the continuous variables followed a normal distribution, they were summarized as medians with interquartile ranges (Q1, Q3), and the Kruskal-Wallis test was used for pairwise comparisons.

To assess the associations between endometrial preparation protocols and pregnancy and obstetric-neonatal outcomes, logistic regression models were used to estimate crude odds ratios (OR) and adjusted odds ratios (AOR) with corresponding 95% confidence intervals (CIs). Univariate logistic regressions were initially conducted between each outcome and all covariates to identify the factors influencing outcomes. Statistically significant covariates with *P* < 0.2 were considered in subsequent multivariate logistic regressions, accounting for the exposure of interest and clinically relevant variables. Age, body mass index (BMI), infertility type, and endometrial preparation protocols were included as fixed variables in the multivariate logistic regression analyses. Given the multiple comparisons conducted across different clinical and obstetric-neonatal outcomes, Bonferroni correction was applied to adjust for the increased risk of type I errors due to multiple hypothesis testing. Both crude and adjusted ORs were reported alongside their corresponding 95% CIs to assess the strength of the associations. Statistical significance was determined with two-sided *P* values, and a threshold of *P* < 0.05 was considered statistically significant for all tests. The sample size was calculated using R based on differences in the incidence of preeclampsia (3.7% versus 11.8%) and HDP (2.8% versus 11.4%) between the NC and AC groups [43]. To provide a two-tailed significance level of 0.05 and a power of 80%, 116 participants were required in the natural cycle group.

## Results

### Demographic characteristics of the study population

The study population comprised 2671 women with thin endometrium (endometrial thickness < 8 mm) who fulfilled the inclusion and exclusion criteria. Respectively, they were grouped by different endometrial preparation protocols for frozen embryo transfer: 1908 (71.43%) patients in the AC group, 506 (18.94%) in the GnRH-a + AC group and 257 (9.62%) in the NC group. Table 1 gives an overview of the characteristics of all patients included. There were significant differences (*P* < 0.05) in antral follicle count (AFC), anti-Müllerian hormone (AMH), cause of infertility, maternal age at oocyte retrieval, and FET among the three groups. As for cycle characteristics in Table 1, gonadotropin dose, number of retrieved oocytes, and MII oocytes significantly differed across the study groups (*P* < 0.05). Similarly, demographic and cycle characteristics of 1104 patients (endometrial thickness < 7 mm) were exhibited in Supplemental Table 1.

**Table 1:** General characteristics and pregnancy outcomes of patients undergoing different endometrial preparation protocols

Variable	Endometrial preparation protocols			*P*
	AC (*N* = 1908)	GnRH-a + AC (*N* = 506)	NC (*N* = 257)	
Maternal age at oocyte retrieval, years	33 (30, 37)^a^	34 (31, 37)^b^	34 (30, 38)^b^	< 0.001^3*^
BMI, kg/m^2^	21.48 (19.92, 23.44)	21.48 (19.98, 23.23)	21.23 (19.80, 23.26)	0.467^3^
Baseline FSH, IU/L	7.38 (6.21, 8.89)	7.56 (6.40, 9.21)	7.57 (6.35, 9.06)	0.212^3^
AFC	11 (7, 17)^a^	10 (6, 15)^b^	9 (6, 13)^b^	< 0.001^3*^
AMH, ng/ml	3.00 (1.55, 5.39)^a^	2.56 (1.31, 4.66)^b^	2.28 (1.29, 3.89)^b^	< 0.001^3*^
Duration of infertility, years	2 (1, 4)	2 (1, 4)	2 (1, 4)	0.379^3^
Infertility type				0.553^1^
Primary infertility	882 (46.2%)	222 (43.9%)	113 (44.0%)	
Second infertility	1026 (53.8%)	284 (56.1%)	144 (56.0%)	
Cause of infertility				< 0.001^2*^
Tubal factor	628 (32.9%)^a^	195 (38.5%)^a^	83 (32.3%)^a^	
Male factor	219 (11.5%)^a^	26 (5.1%)^b^	40 (15.6%)^a^	
Diminished ovarian reserve	259 (13.6%)^a^	93 (18.4%)^b^	42 (16.3%)^a, b^	
Ovulatory disorders	159 (8.3%)^a^	29 (5.7%)^a^	0 (0.0%)^b^	
Endometriosis	37 (1.9%)^a^	31 (6.1%)^b^	3 (1.2%)^a^	
Unexplained	93 (4.9%)^a, b^	14 (2.8%)^b^	17 (6.6%)^a^	
Uterine	139 (7.3%)^a^	26 (5.1%)^a^	23 (8.9%)^a^	
Mixed	374 (19.6%)^a^	92 (18.2%)^a^	49 (19.1%)^a^	
COH protocol				0.105^1^
Depot GnRH-a	224 (11.7%)	52 (10.3%)	24 (9.3%)	
Long GnRH-a	581 (30.5%)	148 (29.2%)	60 (23.3%)	
GnRH antagonist	802 (42.0%)	214 (42.3%)	121 (47.1%)	
Other protocols	301 (15.8%)	92 (18.2%)	52 (20.2%)	
Fertilization method				0.640^1^
IVF	1414 (74.1%)	383 (75.7%)	188 (73.2%)	
ICSI	422 (22.1%)	100 (19.8%)	56 (21.8%)	
IVF + RICSI	72 (3.8%)	23 (4.5%)	13 (5.1%)	
Gonadotrophin duration, days	10 (9, 11)	10 (9, 11)	9 (8, 11)	0.149^3^
Gonadotrophin dose, IU	2400 (1800, 2925)^a^	2550 (1995, 3150)^b^	2475 (1950, 3075)^a, b^	0.008^3*^
No. of oocytes retrieved	11 (7, 17)^a^	10 (7, 15)^b^	10 (6, 15)^b^	0.001^3*^
No. of MⅡ oocytes	10 (6, 15)^a^	9 (5, 13)^b^	9 (5, 13)^a, b^	0.007^3*^
No. of 2PN	7 (4, 10)	7 (4, 9)	6 (4, 10)	0.265^3^
Normal fertilization rate	0.73 (0.60, 0.85)	0.75 (0.60, 0.87)	0.75 (0.61, 0.89)	0.167^3^
Blastocyst formation rate	0.67 (0.33, 0.86)	0.63 (0.31, 0.86)	0.67 (0.24, 0.86)	0.228^3^
Maternal age at FET, years	33 (30, 37)^a^	35 (32, 38)^b^	35 (32, 39)^b^	< 0.001^3*^
Endometrial thickness, mm	7.1 (6.4, 7.5)	7.0 (6.3, 7.5)	7.0 (6.3, 7.5)	0.426^3^
No. of embryos transferred				0.170^1^
One	1468 (76.9%)	369 (72.9%)	196 (76.3%)	
Two	440 (23.1%)	137 (27.1%)	61 (23.7%)	
Type of embryo transferred				0.487^1^
Day 3 (cleavage stage)	530 (27.8%)	150 (29.6%)	79 (30.7%)	
Day 5/6 (blastocyst stage)	1378 (72.2%)	356 (70.4%)	178 (69.3%)	
Pregnancy outcomes				
Biochemical pregnancy loss	165 (8.6%)^a^	35 (6.9%)^a, b^	10 (3.9%)^b^	0.020^1*^
Clinical pregnancy	691 (36.2%)	178 (35.2%)	87 (33.9%)	0.721^1^
Live birth	512 (26.8%)	128 (25.3%)	71 (27.6%)	0.729^1^
Multiple pregnancies	74 (3.9%)	20 (4.0%)	15 (5.8%)	0.325^1^
Ectopic pregnancy	11 (0.6%)	1 (0.2%)	0 (0.0%)	0.303^2^
Miscarriage	176 (25.5%)	49 (27.5%)	16 (18.4%)	0.262^1^

The data are presented as median (Q1, Q3) or N (%)

*BMI* Body mass index, *FSH* Follicle stimulation hormone, *AFC* Antral follicle count, *AMH* Anti-müllierian hormone, *COH* Controlled ovarian hyperstimulation, *IVF* In-vitro fertilization, *ICSI* Intracytoplasmic sperm injection, *RICSI* Rescue ICSI, *MII* Metaphase II, *PN* Pronucleus, *FET* Frozen embryo transfer, *AC* Artificial cycle, *GnRH-a* Gonadotropin-releasing hormone agonist, *NC* Natural cycle

*P < 0.05 (without Bonferroni correction)

^1^Chi-Square p-value; ^2^Fisher Exact p-value; ^3^Kruskal-Wallis p-value

^a, b^Statistically significant differences between groups after Bonferroni correction. Groups sharing the same letter are not significantly different

### Clinical pregnancy outcomes

Table 1 exhibited the pregnancy outcomes and live birth calculation of patients who underwent different endometrial preparation protocols (endometrial thickness < 8 mm). In the AC group, 691 (36.2%) patients realized clinical pregnancies, and 512 (26.8%) patients achieved live births. Patients in the GnRH-a + AC group reached 178 (35.2%) clinical pregnancies and 128 (25.3%) live births. In the NC group, there were 87 (33.9%) clinical pregnancies and 71 (27.6%) live births. The biochemical pregnancy loss rate in NC group 3.9% (10/257) was significantly lower than that in AC group 8.6% (165/1908) (*P* < 0.05). There were no statistical differences between groups in clinical pregnancy, live birth, miscarriage, multiple pregnancy, and ectopic pregnancy. As the incidence of ectopic pregnancy is so low, the power to detect a small difference was limited.

In Table 2, clinical pregnancy and live birth outcomes were analyzed by multivariate logistic regression model, in which AOR and 95% CI were calculated by adjusting predictors (significance level < 0.2), including maternal age at FET, BMI, cause of infertility, number of retrieved oocytes, infertility type, and duration, etc. Endometrial preparation protocol and pregnancy outcome were no association founded, except for biochemical pregnancy loss in the NC referred to AC (OR [95%CI]: 0.428 [0.223,0.821]; AOR [95%CI]: 0.444 [0.230,0.856]).

**Table 2:** Crude and adjusted odds ratios of pregnancy outcomes

Variable	AC vs. NC		AC vs. GnRH-a + AC		NC vs. GnRH-a + AC	
	Crude OR (95% CI)	Adjusted OR (95% CI)	Crude OR (95% CI)	Adjusted OR (95% CI)	Crude OR (95% CI)	Adjusted OR (95% CI)
Biochemical pregnancy loss ^a^	**0.428 (0.223**,**0.821)**	**0.444 (0.230**,**0.856)**	0.785 (0.538,1.146)	0.795 (0.541,1.170)	1.835 (0.894,3.769)	1.791 (0.866,3.702)
Clinical pregnancy ^b^	0.901 (0.685,1.186)	1.067 (0.795,1.434)	0.956 (0.779,1.173)	1.133 (0.907,1.415)	1.060 (0.773,1.455)	1.061 (0.755,1.492)
Live birth ^b^	1.041 (0.778,1.393)	1.264 (0.921,1.735)	0.923 (0.738,1.156)	1.117 (0.876,1.426)	0.887 (0.632,1.245)	0.884 (0.611,1.277)
Multiple pregnancy ^c^	1.536 (0.868,2.719)	1.775 (0.941,3.349)	1.020 (0.616,1.688)	1.022 (0.593,1.762)	0.664 (0.334,1.320)	0.576 (0.271,1.224)
Ectopic pregnancy ^d^	NA	NA	0.341 (0.044,2.651)	1.141 (0.682,1.909)	NA	NA
Miscarriage ^e^	0.659 (0.373,1.165)	0.616 (0.342,1.111)	1.111 (0.767,1.610)	0.955 (0.645,1.413)	1.686 (0.894,3.179)	1.549 (0.799,3.002)
	Referred to AC		Referred to AC		Referred to NC	

Odds ratios (ORs) and 95% confidence intervals (CIs) are based on the univariate analysis. Adjusted odds ratios (AORs) and 95% CIs are based on the multiple logistic regression model

NA: Insufficient data for statistical analysis

*AC* Artificial cycle, *NC* Natural cycle, *GnRH-a* Gonadotropin-releasing hormone agonist, *OR* Odds ratios, *CI* Confidence interval, *FET* Frozen embryo transfer, *BMI* Body mass index, *COH* Controlled ovarian hyperstimulation

^a^Adjusted for maternal age at FET, BMI, cause of infertility, number of retrieved oocytes, infertility type, duration of infertility, and controlled ovarian hyperstimulation (COH) protocol

^b^Adjusted for maternal age at FET, BMI, cause of infertility, fertilization method, number of retrieved oocytes, COH protocol, type of embryos transferred, and endometrial thickness

^c^Adjusted for maternal age at FET, BMI, cause of infertility, number of retrieved oocytes, COH protocol, endometrial thickness, and number of embryos transferred

^d^Adjusted for maternal age at FET, BMI, cause of infertility, and number of retrieved oocytes

^e^Adjusted for maternal age at FET, BMI, cause of infertility, fertilization method, number of retrieved oocytes, infertility type, COH protocol, number of embryos transferred, and endometrial thickness

In patients with endometrial thickness < 7 mm, there was no significant difference in pregnancy outcomes and live birth rate among the three groups (Supplemental Tables 1–2).

### Singleton obstetric-neonatal outcomes

Demographic characteristics of patients with singleton live birth were detailed in Table 3 (endometrial thickness < 8 mm). A total of 652 patients were included, with 473 (72.55%) in the AC group, 117 (17.94%) in the GnRH-a + AC group, and 62 (9.51%) in the NC group. AMH level was prominently different (*P* < 0.05) in the NC group compared to the AC group. For AC, GnRH-a + AC, and NC groups, the median birth weights were 3.25 kg, 3.20 kg, and 3.25 kg, and the median gestational ages were 38.7 weeks, 38.6 weeks, and 38.6 weeks. Singleton obstetric-neonatal outcomes of patients who underwent disparate endometrial preparation protocols were summarized in Table 3 (endometrial thickness < 8 mm). There was no significant difference in cesarean delivery, low birth weight, macrosomia, small for gestational age, large for gestational age, preterm birth, gestational diabetes mellitus, hypertensive disorder of pregnancy, and fetal malformation in comparison between groups.

**Table 3:** General characteristics and obstetric-neonatal outcomes of patients with singleton live births

Variable	Endometrial preparation protocols			*P*
	AC (*N* = 473)	GnRH-a + AC (*N* = 117)	NC (*N* = 62)	
Maternal age at oocyte retrieval, years	31 (29, 34)	32 (30, 35)	32 (29, 36)	0.248^3^
BMI, kg/m^2^	21.32 (19.68, 23.44)	20.83 (19.65, 23.19)	21.14 (20.28, 24.08)	0.371^3^
Baseline FSH, IU/L	7.23 (6.05, 8.52)	7.32 (6.28, 8.29)	7.39 (5.99, 8.39)	0.627^3^
AFC	12 (8, 19)	10 (7, 18)	7 (10, 16)	0.101^3^
AMH, ng/ml	3.41 (2.01, 5.86)^a^	2.88 (1.65, 5.18)^a, b^	2.29 (1.45, 4.25)^b^	0.005^3*^
Duration of infertility, years	2 (1, 4)	2 (1, 4)	2 (1.5, 4)	0.361^3^
Infertility type				0.037^1*^
Primary infertility	236 (49.9%)^a, b^	46 (39.3%)^b^	36 (58.1%)^a^	
Second infertility	237 (50.1%)^a, b^	71 (60.7%)^b^	26 (41.9%)^a^	
Cause of infertility				< 0.001^2*^
Tubal factor	153 (32.3%)^a^	44 (37.6%)^a^	25 (37.3%)^a^	
Male factor	74 (15.6%)^a^	7 (6.0%)^b^	12 (19.4%)^a^	
Diminished ovarian reserve	32 (6.8%)^a^	11 (9.4%)^a^	9 (14.5%)^a^	
Ovulatory disorders	61 (12.9%)^a^	10 (8.5%)^a^	0 (0.0%)^b^	
Endometriosis	7 (1.5%)^a^	10 (8.5%)^b^	0 (0.0%)^a, b^	
Unexplained	33 (7.0%)^a^	4 (3.4%)^a^	7 (11.3%)^a^	
Uterine	27 (5.7%)^a^	7 (6.0%)^a^	3 (4.8%)^a^	
Mixed	86 (18.2%)^a^	24 (20.5%)^a^	6 (9.7%)^a^	
COH protocol				0.261^1^
Depot GnRH-a	52 (11.0%)	16 (13.7%)	8 (12.9%)	
Long GnRH-a	165 (34.9%)	39 (33.3%)	12 (19.4%)	
GnRH antagonist	207 (43.8%)	52 (44.4%)	32 (51.6%)	
Other protocols	49 (10.4%)	10 (8.5%)	10 (16.1%)	
Fertilization method				0.789^2^
IVF	344 (72.7%)	85 (72.6%)	45 (72.6%)	
ICSI	118 (24.9%)	27 (23.1%)	16 (25.8%)	
IVF + RICSI	11 (2.3%)	5 (4.3%)	1 (1.6%)	
Gonadotrophin duration, days	10 (9, 11)	10 (9, 11)	10 (8, 10)	0.093^3^
Gonadotrophin dose, IU	2250 (1650, 2785)^a^	2550 (1950, 3135)^b^	2300 (1850, 2700)^a, b^	0.046^3*^
No. of oocytes retrieved	14 (8, 19)	11 (8, 16)	11 (7, 18)	0.103^3^
No. of MⅡ oocytes	11 (7, 17)	9 (6, 15)	6 (10, 16)	0.055^3^
No. of 2PN	8 (5, 12)	7 (5, 10)	5 (7, 11)	0.279^3^
Normal fertilization rate	0.75 (0.62, 0.85)	0.77 (0.67, 0.88)	0.75 (0.60, 0.85)	0.556^3^
Blastocyst formation rate	0.73 (0.50, 0.89)	0.71 (0.35, 0.86)	0.75 (0.49, 1.00)	0.538^3^
Maternal age at FET, years	32 (29, 35)	33 (30, 35)	33 (30, 37)	0.079^3^
Endometrial thickness, mm	7.2 (6.7, 7.6)	7.3 (6.8, 7.6)	7.2 (6.5, 7.6)	0.897^3^
No. of embryos transferred				0.357^1^
One	398 (84.1%)	92 (78.6%)	52 (83.9%)	
Two	75 (15.9%)	25 (21.4%)	10 (16.1%)	
Type of embryo transferred				0.181^1^
Day 3 (cleavage stage)	63 (13.3%)	19 (16.2%)	4 (6.5%)	
Day 5/6 (blastocyst stage)	410 (86.7%)	98 (83.8%)	58 (93.5%)	
Obstetric-neonatal outcomes				
Delivery mode				0.064^1^
Cesarean delivery	414 (87.5%)	102 (87.2%)	48 (77.4%)	
Natural labor	59 (12.5%)	15 (12.8%)	14 (22.6%)	
Gender				0.624^1^
Male	240 (50.7%)	65 (55.6%)	34 (54.8%)	
Female	233 (49.3%)	52 (44.4%)	28 (45.2%)	
Gestational age, weeks	38.7 (37.9, 39.3)	38.6 (37.9, 39.1)	38.6 (37.9, 39.1)	0.991^3^
Birth weight, kg	3.25 (3.00, 3.54)	3.20 (2.94, 3.50)	3.25 (3.00, 3.60)	0.608^3^
Low birth weight < 2, 500 g	40 (8.5%)	7 (6.0%)	4 (6.5%)	0.620^2^
Macrosomia > 4, 000 g	23 (4.9%)	5 (4.3%)	1 (1.6%)	0.521^2^
Small for gestational age	21 (4.4%)	7 (6.0%)	1 (1.6%)	0.421^2^
Large for gestational age	73 (15.4%)	12 (10.3%)	6 (9.7%)	0.208^1^
Preterm birth	63 (13.3%)	15 (12.8%)	10 (16.1%)	0.813^1^
Gestational diabetes mellitus	14 (3.0%)	3 (2.6%)	1 (1.6%)	0.872^2^
Hypertensive disorders of pregnancy	17 (3.6%)	5 (4.3%)	5 (8.1%)	0.251^2^
Placenta previa	12 (2.5%)	4 (3.4%)	1 (1.6%)	0.730^2^
Fetal malformation	2 (0.4%)	2 (1.7%)	0 (0.0%)	0.197^2^

*AC* Artificial cycle, *GnRH-a* Gonadotropin-releasing hormone agonist, *NC* Natural cycle, *FET* Frozen embryo transfer, *BMI* Body mass index, *FSH* Follicle stimulation hormone, *AFC* Antral follicle count, *AMH* Anti-müllierian hormone, *COH* Controlled ovarian hyperstimulation, *PN* Pronucleus

* P < 0.05 (without Bonferroni correction)

^1^Chi-Square p-value; ^2^Fisher Exact p-value; ^3^Kruskal-Wallis p-value

^a, b^Statistically significant differences between groups after Bonferroni correction. Groups sharing the same letter are not significantly different

Using the method previously stated, adjusted odds ratios and 95%CI were calculated to analyze the association between FET endometrial preparation protocols and singleton obstetric-neonatal outcomes. All the outcome variables were still retained. No significant difference was found after univariate and multivariate regression analyses (Table 4). In Supplemental Tables 3–4, the same study was conducted in patients with endometrial thickness < 7 mm, showing no significant difference in obstetric-neonatal outcomes across the three groups.

**Table 4:** Crude and adjusted odds ratios of obstetric-neonatal outcomes

Variable	AC vs. NC		AC vs. GnRH-a + AC		NC vs. GnRH-a + AC	
	Crude OR (95% CI)	Adjusted OR (95% CI)	Crude OR (95% CI)	Adjusted OR (95% CI)	Crude OR (95% CI)	Adjusted OR (95% CI)
Low birth weight ^a^	0.747 (0.258, 2.163)	0.775 (0.259, 2.316)	0.689 (0.300, 1.580)	0.594 (0.250, 1.410)	0.923 (0.259, 3.282)	0.766 (0.206, 2.856)
Macrosomia ^b^	0.321 (0.043, 2.418)	0.284 (0.036, 2.254)	0.873 (0.325, 2.348)	0.880 (0.311, 2.486)	2.723 (0.311, 23.840)	3.102 (0.332, 28.984)
SGA ^c^	0.353 (0.047, 2.670)	0.365 (0.047, 2.813)	1.370 (0.568, 3.304)	1.222 (0.494, 3.021)	3.882 (0.467, 32.292)	3.344 (0.391, 28.611)
LGA ^d^	0.587 (0.244, 1.413)	0.608 (0.247, 1.499)	0.626 (0.328, 1.196)	0.661 (0.336, 1.304)	1.067 (0.380, 2.995)	1.087 (0.371, 3.184)
Preterm birth ^e^	1.242 (0.601, 2.570)	1.293 (0.607, 2.757)	0.950 (0.520, 1.737)	0.797 (0.428, 1.485)	0.765 (0.321, 1.820)	0.616 (0.249, 1.527)
GDM ^f^	0.534 (0.069, 4.132)	0.621 (0.077, 4.995)	0.857 (0.242, 3.033)	0.757 (0.203, 2.828)	1.605 (0.163, 15.764)	1.220 (0.115, 12.970)
HDP ^c^	2.337 (0.831, 6.577)	2.359 (0.798, 6.974)	1.190 (0.430, 3.294)	1.151 (0.410, 3.231)	0.509 (0.142, 1.830)	0.488 (0.129, 1.843)
Placenta previa ^b^	0.626 (0.080, 4.896)	0.589 (0.073, 4.752)	1.351 (0.428, 4.268)	1.547 (0.468, 5.115)	2.159 (0.236, 19.749)	2.629 (0.270, 25.626)
Fetal malformation ^b^	NA	NA	4.070 (0.567, 29.198)	5.443 (0.668, 44.347)	NA	NA
	Referred to AC		Referred to AC		Referred to NC	

Odds ratios (ORs) and 95% confidence intervals (CIs) are based on the univariate analysis. Adjusted odds ratios (AORs) and 95% CIs are based on the multiple logistic regression model

NA: Insufficient data for statistical analysis

*AC* Artificial cycle, *NC* Natural cycle, *GnRH-a* Gonadotropin-releasing hormone agonist, *OR* Odds ratios, *CI* Confidence interval, *SGA* Small for gestational age, *LGA* Large for gestational age, *GDM* Gestational diabetes mellitus, *HDP* Hypertensive disorders of pregnancy, *FET* Frozen embryo transfer, *BMI* Body mass index, *COH* Controlled ovarian hyperstimulation

^a^Adjusted for maternal age at FET, BMI, cause of infertility, duration of infertility, and number of retrieved oocytes

^b^Adjusted for maternal age at FET, BMI, and cause of infertility

^c^Adjusted for maternal age at FET, BMI, cause of infertility, and number of retrieved oocytes

^d^Adjusted for maternal age at FET, BMI, cause of infertility, COH protocol, number of retrieved oocytes, and number of embryo transferred

^e^Adjusted for maternal age at FET, BMI, cause of infertility, infertility type, and number of retrieved oocytes

^f^Adjusted for maternal age at FET, BMI, cause of infertility, and number of embryo transferred

Throughout this analysis, the NC group contained both true natural cycles and modified natural cycles, which were different in HCG trigger and luteal phase support. To minimize the potential confounding effects of such differences, we categorized the true natural cycle and the modified natural cycle into distinct groups and compared both baseline characteristics and pregnancy as well as obstetric-neonatal outcomes. The results are summarized in Supplementary Tables 5–6. It revealed that the pregnancy rate and live birth rate were comparable, and no significant differences were observed in obstetric-neonatal complications.

## Discussion

To our knowledge, this is the first research to compare the obstetric-neonatal outcomes among patients with thin endometrium based on different endometrial preparation regimens: natural cycle versus artificial cycle with or without GnRH-a pretreatment. According to our findings, whether endometrial thickness < 7–8 mm, the three strategies were comparable in terms of pregnancy and obstetric-neonatal outcomes after FET. But the biochemical pregnancy loss was significantly lower in NC than that in AC in patients with endometrial thickness < 8 mm (3.9% versus 8.5%; AOR [95%CI]: 0.444 [0.230, 0.856]). However, the statistical power for these comparisons was limited due to the low incidence of obstetric-neonatal complications in each group, possibly reducing the ability to detect a true difference.

Managing patients with thin endometrium remains an inevitable obstacle to IVF. Many promising treatment regimens to enhance endometrial thickness have recently focused on FET cycles, including growth hormone, intrauterine autologous platelet-rich plasma infusion, tamoxifen, and granulocyte colony-stimulating factor [44–46]. However, these controversial approaches for managing thin endometrium still need to be clarified.

This investigation of thin endometrium found no statistical differences in the incidence of obstetric-neonatal complications among endometrial preparation protocols, which have not been reported before. In the general population, however, it remains uncertain whether the more physiological hormonal environment of NC reduces such complications compared with AC [11, 15, 47–49]. Based on data from a single-center randomized controlled trial of 1428 women, no differences were found among NC, modified NC, and AC, comparable to other literature results [15]. In contrast, a recent systematic review and meta-analysis involving 30 studies and 113,676 patients (NC: *N* = 56, 445; AC: *N* = 57, 231) concluded that singleton deliveries from NC decreased the risk of adverse obstetric-neonatal outcomes compared to AC [11]. The corpora lutea in NC has been estimated to be associated with safer outcomes in FET [50–52]. However, this does not seem to be true regarding thin endometrium. In this present study of thin endometrium, obstetric-neonatal outcomes were not influenced by the different endometrial preparation protocols. This may be because a thin endometrium itself can lead to various obstetric and neonatal complications, including SGA, LBW, HDP, placental disease, and preterm birth [32–35]. These risks are difficult to mitigate through endometrial preparation protocols alone. A cohort study of 1057 deliveries following IVF examined placental pathology [35]. It revealed a reduced placental thickness, a higher rate of placental abruption, and alterations in the frequency of maternal hypoperfusion in the thin endometrium group. Studies have shown that fresh embryo transfer with more corpora lutea has a lower risk of HDP, suggesting that the number of corpora lutea may mediate placental perfusion [9, 10]. Therefore, more intensive interventions may be necessary to enhance obstetric outcomes in patients with thin endometrium. Our team is currently conducting a study on the impact of the number of corpora lutea on the outcome of FET, and the results are worth attention. Another reason for this inconsistency was that obstetric-neonatal complications have decreased in FET cycles due to advancements in IVF technology. The variation between study populations, embryo culture medium, cryoprotectant choice, and slow freezing versus vitrification may contribute to substantial inter-study heterogeneity.

Regarding pregnancy outcomes, including clinical pregnancy and live birth rate, previous studies have explored which endometrial preparation protocol could create a more receptive uterine environment to embryo implantation. Similar to our research, numerous studies have found no superiority in which treatment regimen provides the best pregnancy rates and clinical outcomes in general populations [12, 13, 15]. Our center’s three most common endometrial preparation strategies (NC, AC, and GnRH-a + AC) failed to offer a satisfactory solution for the challenging problem of thin endometrium. However, three studies have been conducted on the relative effectiveness of stimulated protocols in women with thin endometrium, which seemed promising. In a recent historical cohort study of thin endometrium, 129 women received the AC regimen, and 249 received the ovarian induction following GnRH agonist pretreatment [37]. The investigators found that clinical pregnancy and live birth rates in FET cycles were significantly higher in the stimulated cycles, similar to a case report [38]. Another case-control study involved 40 women for the stimulated protocol and 40 age-matched women for the AC protocol in patients with thin endometrium. The clinical pregnancy rate was 35% and 12.5%, respectively (*P* = 0.017) [39]. These studies showed the beneficial effects of stimulated cycles and deserved further study.

In this study, the biochemical pregnancy loss rate was lower in NC (3.9%) than that in AC (8.6%) and GnRH-a + AC (6.9%) (*P* = 0.02). The risk of pregnancy loss was also lower in NC but not significant (NC: 18.4%; AC: 25.5%; GnRH-a + AC: 27.5%). As indicated by previous research, ovulatory disorders, mainly caused by PCOS, was associated with biochemical pregnancy loss [53]. In this research, we performed a multivariate logistic regression analysis, adjusting for the relevant covariates including the cause of infertility. The difference in biochemical pregnancy loss rates between NC and AC remained statistically significant. Therefore, for patients with thin endometrium and normal ovulatory function, using the natural cycle for FET may help reduce the rate of biochemical pregnancy loss. Interestingly, we found a higher biochemical pregnancy loss rate but a similar live birth rate in the AC group than the NC group. A multicenter historical cohort study, involving AC (*N* = 8139) and NC (*N* = 3126), concluded that AC was associated with higher early pregnancy loss rates (AC: 36.5%; NC: 25.6%; AOR [95% CI]: 1.63 [1.35, 1.97]) and lower live birth rates, but biochemical pregnancy loss rates were similar [54]. A retrospective study showed significantly higher early pregnancy loss rates (34.2% versus 56.9%) and lower live birth rates (59.7% versus 29.1%) in NC compared to stimulated cycles [55]. Another retrospective study involving 4470 cycles found comparable clinical pregnancy and live birth rates across all protocols, though AC exhibited higher biochemical pregnancy loss rates [56]. In contrast, a randomised controlled trial [13] involving women with normal ovulatory function revealed no significant differences in live birth or pregnancy loss rates between NC, modified NC and AC. These inconsistencies may stem from multiple factors: firstly, differences in study design, where RCTs control for bias through randomisation but may lack generalisability, while observational studies better reflect real-world settings yet remain susceptible to confounding factors; secondly, variations in outcome definitions and measurement timepoints, such as distinguishing early pregnancy loss from biochemical pregnancy loss; thirdly, variations in protocol execution details, such as drug dosages or monitoring frequencies; fourthly, the predominantly low quality of evidence, as noted in systematic reviews [14], necessitates further high-quality RCTs to clarify these findings.

Besides, we provided separate analyses of pregnancy and obstetric-neonatal outcomes from AC with or without GnRH-a. As the AC is currently the most common method of endometrial preparation, finding out populations benefiting from pretreatment with 3.75 mg of GnRH-a was clinically valuable. Molecular mechanism studies have shown that high-dose, long-acting GnRH-a benefits the expression of endometrial receptivity indicators, such as HOXA10, MEIS1, and LIF, improving endometrial receptivity [57]. Other studies found that GnRH-a enhanced the number of pinocytosis and the expression of integrins [58, 59]. Therefore, it was reported that the depot GnRH-a protocol with downregulation of 3.75 mg GnRH-a could improve the outcome of embryo transfer in the general population [57] as well as in patients with a thin endometrium [60]. Besides, long-acting GnRH-a can effectively prevent the subsequent occurrence of a dominant follicle in AC. However, our study found that thin endometrium does not seem to require GnRH-a pretreatment, which did not improve the clinical pregnancy and live birth rate, but neither did the pregnancy loss nor obstetric-neonatal complications. Moreover, additional GnRH-a treatment will delay the time for patients to obtain live births and increase the financial burden on patients. One possible explanation may be that the GnRH-a pretreatment in our trial was only one month. Only one small-sample observation historical study has been conducted on women with thin endometrium, and the results showed a promotion in clinical outcomes when treating patients for two months [61].

Our research has some limitations. The primary constraints of this study stem from its single-centered retrospective historical design and inherent selection bias. However, we included a relatively large sample size and adjusted for confounding variables through multivariate logistic regression to enhance the reliability of our results. Nevertheless, some confounding variables that were overlooked exist inevitably. In addition, the generalizability of our findings may be restricted to the exclusion of stimulated cycles in this study, especially given their potential benefits for thin endometrium, as referenced in prior studies. The small cohort of 40 patients in stimulated cycles with thin endometrium was inadequate for inclusion in our analysis. In our center, natural cycles and artificial cycles with or without GnRH agonist are the most common protocols. The incidence of thin endometrium is low. In order to ensure sufficient enrollment and statistical significance, we screened 2671 patients who met the inclusion criteria from 29,338 FET cycles. As these three common endometrial preparation regimens had no priority in patients with thin endometrium, our team is conducting a study on ovulation induction to induce multiple corpora lutea in the hope of bringing significant benefits.

## Conclusions

The natural cycle might decrease the biochemical pregnancy loss, referred to as the AC cycle for patients with endometrial thickness < 8 mm. The live birth rate and obstetric outcomes were not affected beneficially or detrimentally by endometrial preparation strategies in natural cycles, artificial cycles, or GnRH-a artificial cycle for thin endometrium (endometrial thickness < 7–8 mm on the first day of progesterone administration) women undergoing FET.

## Supplementary Information

Supplementary Material 1. Supplemental Table 1. General characteristics and pregnancy outcomes of patients undergoing different endometrial preparation protocols (endometrial thickness < 7 mm). Supplemental Table 2. Crude and adjusted odds ratios of pregnancy outcomes (endometrial thickness < 7 mm). Supplemental Table 3. General characteristics and obstetric-neonatal outcomes of patients with singleton live births (endometrial thickness < 7 mm). Supplemental Table 4. Crude and adjusted odds ratios of obstetric-neonatal outcomes (endometrial thickness < 7 mm). Supplemental Table 5. General characteristics of patients undergoing true natural cycles and modified natural cycles (endometrial thickness < 8 mm). Supplemental Table 6. Crude and adjusted odds ratios of pregnancy and obstetric-neonatal outcomes in true natural cycles and modified natural cycles (endometrial thickness < 8 mm)

## Data Availability

Data will be shared with other researchers for further meta-analysis following article publication upon request by email to the corresponding author.

## Abbreviations

FET: Frozen embryo transfer
NC: Natural cycle
AC: Artificial cycle
GnRH-a: Gonadotropin-releasing hormone agonist
IVF: In-vitro fertilization
HDP: Hypertensive disorders of pregnancy
PCOS: Polycystic ovary syndrome
HCG: Human chorionic gonadotropin
CPR: Clinical pregnancy rate
LBR: Live birth rate
LBW: Low birth weight
SGA: Small-for-gestational-age
LGA: Large-for-gestational-age
OR: Odds ratios
AOR: Adjusted odds ratios
CIs: Confidence intervals
BMI: Body mass index
AFC: Antral follicle count
AMH: Anti-müllierian hormone

## References

[1] Sunderam S, Kissin DM, Zhang Y, Jewett A, Boulet SL, Warner L, et al. Assisted reproductive technology surveillance - United States, 2018. MMWR Surveill Summ. 2022;71(4):1–19. 35176012 10.15585/mmwr.ss7104a1 PMC8865855

[2] Bai F, Wang DY, Fan YJ, Qiu J, Wang L, Dai Y, et al. Assisted reproductive technology service availability, efficacy and safety in mainland china: 2016. Hum Reprod. 2020;35(2):446–52. 32020190 10.1093/humrep/dez245

[3] Wyns C, De Geyter C, Calhaz-Jorge C, Kupka MS, Motrenko T, Smeenk J, et al. ART in Europe, 2018: results generated from European registries by ESHRE. Hum Reprod Open. 2022;2022(3):hoac022. 35795850 10.1093/hropen/hoac022 PMC9252765

[4] Banker M, Dyer S, Chambers GM, Ishihara O, Kupka M, de Mouzon J, et al. International committee for monitoring assisted reproductive technologies (ICMART): world report on assisted reproductive technologies, 2013. Fertil Steril. 2021;116(3):741–56. 33926722 10.1016/j.fertnstert.2021.03.039

[5] Chambers GM, Dyer S, Zegers-Hochschild F, de Mouzon J, Ishihara O, Banker M, et al. International committee for monitoring assisted reproductive technologies world report: assisted reproductive technology, 2014. Hum Reprod. 2021;36(11):2921–34. 34601605 10.1093/humrep/deab198

[6] Rienzi L, Gracia C, Maggiulli R, LaBarbera AR, Kaser DJ, Ubaldi FM, et al. Oocyte, embryo and blastocyst cryopreservation in ART: systematic review and meta-analysis comparing slow-freezing versus vitrification to produce evidence for the development of global guidance. Hum Reprod Update. 2017;23(2):139–55. 27827818 10.1093/humupd/dmw038 PMC5850862

[7] Wong KM, Mastenbroek S, Repping S. Cryopreservation of human embryos and its contribution to in vitro fertilization success rates. Fertil Steril. 2014;102(1):19–26. 24890275 10.1016/j.fertnstert.2014.05.027

[8] Wei D, Liu JY, Sun Y, Shi Y, Zhang B, Liu JQ, et al. Frozen versus fresh single blastocyst transfer in ovulatory women: a multicentre, randomised controlled trial. Lancet. 2019;393(10178):1310–8. 30827784 10.1016/S0140-6736(18)32843-5

[9] Wiegel RE, Jan Danser AH, Steegers-Theunissen RPM, Laven JSE, Willemsen SP, Baker VL, et al. Determinants of maternal Renin-Angiotensin-Aldosterone-System activation in early pregnancy: insights from 2 cohorts. J Clin Endocrinol Metab. 2020;105(11):3505–17. 32853347 10.1210/clinem/dgaa582 PMC7494245

[10] Koerts JJ, Voskamp LW, Rousian M, Steegers-Theunissen RPM, Wiegel RE. Impact of corpus luteum number on maternal pregnancy and birth outcomes: the Rotterdam periconception cohort. Fertil Steril. 2025;123(6):1039–50. 39644989 10.1016/j.fertnstert.2024.12.002

[11] Zaat TR, Kostova EB, Korsen P, Showell MG, Mol F, van Wely M. Obstetric and neonatal outcomes after natural versus artificial cycle frozen embryo transfer and the role of luteal phase support: a systematic review and meta-analysis. Hum Reprod Update. 2023;29(5):634–54. 37172270 10.1093/humupd/dmad011 PMC10477943

[12] Roelens C, Blockeel C. Impact of different endometrial preparation protocols before frozen embryo transfer on pregnancy outcomes: a review. Fertil Steril. 2022;118(5):820–7. 36273850 10.1016/j.fertnstert.2022.09.003

[13] Ho VNA, Pham TD, Nguyen NT, Wang R, Norman RJ, Mol BW, et al. Livebirth rate after one frozen embryo transfer in ovulatory women starting with natural, modified natural, or artificial endometrial preparation in Viet nam: an open-label randomised controlled trial. Lancet. 2024;404(10449):266–75. 38944045 10.1016/S0140-6736(24)00756-6

[14] Ghobara T, Gelbaya TA, Ayeleke RO. Cycle regimens for frozen-thawed embryo transfer. Cochrane Database Syst Rev. 2017;7(7):Cd003414. 28675921 10.1002/14651858.CD003414.pub3 PMC6483463

[15] Busnelli A, Schirripa I, Fedele F, Bulfoni A, Levi-Setti PE. Obstetric and perinatal outcomes following programmed compared to natural frozen-thawed embryo transfer cycles: a systematic review and meta-analysis. Hum Reprod. 2022;37(7):1619–41. 35553678 10.1093/humrep/deac073

[16] Yuan Y, Chang Q, Wen Y, Gao J, Huang S, Xu Y, et al. Letrozole during frozen embryo transfer in women with polycystic ovarian syndrome: a randomized controlled trial. Obstet Gynecol. 2023;142(5):1087–95. 37708500 10.1097/AOG.0000000000005367

[17] Salemi S, Yahyaei A, Vesali S, Ghaffari F. Endometrial preparation for vitrified-warmed embryo transfer with or without GnRH-agonist pre-treatment in patients with polycystic ovary syndrome: a randomized controlled trial. Reprod Biomed Online. 2021;43(3):446–52. 34340936 10.1016/j.rbmo.2021.06.006

[18] Li J, Lin Z, Mo S, Wang S, Li Y, Shi Q. Pretreatment with or without GnRH-agonist before frozen-thawed embryo transfer in patients with PCOS: a systematic review and meta-analysis. J Ovarian Res. 2024;17(1):130. 38907340 10.1186/s13048-024-01410-7 PMC11193291

[19] Niu Z, Chen Q, Sun Y, Feng Y. Long-term pituitary downregulation before frozen embryo transfer could improve pregnancy outcomes in women with adenomyosis. Gynecol Endocrinol. 2013;29(12):1026–30. 24006906 10.3109/09513590.2013.824960

[20] Guo Y, Fang Z, Yu L, Sun X, Li F, Jin L. Which endometrial preparation protocol provides better pregnancy and perinatal outcomes for endometriosis patients in frozen-thawed embryo transfer cycles? A retrospective study on 1413 patients. J Ovarian Res. 2023;16(1):7. 36624470 10.1186/s13048-023-01095-4 PMC9830850

[21] Magnusson Å, Hanevik HI, Laivuori H, Loft A, Piltonen T, Pinborg A, et al. Endometrial preparation protocols prior to frozen embryo transfer - convenience or safety? Reprod Biomed Online. 2024;48(1):103587. 37949762 10.1016/j.rbmo.2023.103587

[22] Polyzos NP. Endometrial preparation protocols for frozen embryo transfer: risk assessment and individualized management. Hum Reprod. 2025;deaf149. 10.1093/humrep/deaf149 40796314

[23] Hamze H, Alameh W, Hemmings R, Jamal W, Banan A, Sylvestre C. The science of frozen embryo transfer, is modified natural cycle better? Gynecol Endocrinol. 2025;41(1):2533481. 40671602 10.1080/09513590.2025.2533481

[24] Liu X, Li W, Wen W, Wang T, Wang T, Sun T, et al. Natural cycle versus hormone replacement therapy as endometrial preparation in ovulatory women undergoing frozen-thawed embryo transfer: the compete open-label randomized controlled trial. PLoS Med. 2025;22(6):e1004630. 40561125 10.1371/journal.pmed.1004630 PMC12193059

[25] Huang J, Liao Y, Xia L, Wu H, Liu Z, Lin J, et al. Impact of endometrial preparation protocols on pregnancy outcomes in patients with unexplained recurrent implantation failure undergoing frozen embryo transfer. Ultrasound Obstet Gynecol. 2025;65(5):633–40. 40150934 10.1002/uog.29209

[26] Guo Z, Chu R, Zhang L, Yu Q, Yan L, Ma J. Fresh versus frozen embryo transfer in women with thin endometrium: a retrospective cohort study. Ann Transl Med. 2020;8(21):1435. 33313180 10.21037/atm-20-3230 PMC7723638

[27] Stentz N, Devine K. Through thick and thin: time to stop worrying about endometrial thickness? Fertil Steril. 2021;116(1):71–2. 34052013 10.1016/j.fertnstert.2021.05.072

[28] Liu KE, Hartman M, Hartman A, Luo ZC, Mahutte N. The impact of a thin endometrial lining on fresh and frozen-thaw IVF outcomes: an analysis of over 40 000 embryo transfers. Hum Reprod. 2018;33(10):1883–8. 30239738 10.1093/humrep/dey281 PMC6145412

[29] Kasius A, Smit JG, Torrance HL, Eijkemans MJ, Mol BW, Opmeer BC, Broekmans FJ. Endometrial thickness and pregnancy rates after IVF: a systematic review and meta-analysis. Hum Reprod Update. 2014;20(4):530–41. 24664156 10.1093/humupd/dmu011

[30] Mahutte N, Hartman M, Meng L, Lanes A, Luo ZC, Liu KE. Optimal endometrial thickness in fresh and frozen-thaw *in vitro* fertilization cycles: an analysis of live birth rates from 96,000 autologous embryo transfers. Fertil Steril. 2022;117(4):792–800. 35109980 10.1016/j.fertnstert.2021.12.025

[31] Gallos ID, Khairy M, Chu J, Rajkhowa M, Tobias A, Campbell A, et al. Optimal endometrial thickness to maximize live births and minimize pregnancy losses: analysis of 25,767 fresh embryo transfers. Reprod Biomed Online. 2018;37(5):542–8. 30366837 10.1016/j.rbmo.2018.08.025

[32] Fang Z, Huang J, Mao J, Yu L, Wang X. Effect of endometrial thickness on obstetric and neonatal outcomes in assisted reproduction: a systematic review and meta-analysis. Reprod Biol Endocrinol. 2023;21(1):55. 37312205 10.1186/s12958-023-01105-6 PMC10262454

[33] Ganer Herman H, Volodarsky-Perel A, Ton Nu TN, Machado-Gedeon A, Cui Y, Shaul J, Dahan MH. Pregnancy complications and placental histology following embryo transfer with a thinner endometrium. Hum Reprod. 2022;37(8):1739–45. 35771669 10.1093/humrep/deac148

[34] Guo Z, Xu X, Zhang L, Zhang L, Yan L, Ma J. Endometrial thickness is associated with incidence of small-for-gestational-age infants in fresh in vitro fertilization-intracytoplasmic sperm injection and embryo transfer cycles. Fertil Steril. 2020;113(4):745–52. 32147172 10.1016/j.fertnstert.2019.12.014

[35] Oron G, Hiersch L, Rona S, Prag-Rosenberg R, Sapir O, Tuttnauer-Hamburger M, et al. Endometrial thickness of less than 7.5 mm is associated with obstetric complications in fresh IVF cycles: a retrospective cohort study. Reprod Biomed Online. 2018;37(3):341–8. 30146441 10.1016/j.rbmo.2018.05.013

[36] Liu KE, Hartman M, Hartman A. Management of thin endometrium in assisted reproduction: a clinical practice guideline from the Canadian fertility and andrology society. Reprod Biomed Online. 2019;39(1):49–62. 31029557 10.1016/j.rbmo.2019.02.013

[37] Wang P, Yang H, Chen Z, Chen Y, Jin C, Yu R, et al. Agonist long protocol improves outcomes of vitrified-warmed embryo transfer in repeatedly thin endometrium. Reprod Biomed Online. 2023;46(3):527–35. 36604214 10.1016/j.rbmo.2022.12.003

[38] Ali J, Magray S, Ahmed E, Talo S. A case report of successful live pregnancy following embryo transfer in a thin endometrium. Cureus. 2024;16(8):e66363. 39246935 10.7759/cureus.66363 PMC11378461

[39] Nagaraja N, Poddar SD, Rai S, Verma V, Abhisheka K, Khurana A. Improved pregnancy outcomes and endometrial receptivity by thawed frozen embryo transfer in mildly stimulated cycles with letrozole combined with estrogen in women with unresponsive thin endometrium compared to standard endometrial preparation with estrogen alone: a retrospective study. J Obstet Gynaecol India. 2023;73(4):351–7. 37701079 10.1007/s13224-023-01813-4 PMC10492730

[40] Xia L, Tian L, Zhang S, Huang J, Wu Q. Hormonal replacement treatment for Frozen-Thawed embryo transfer with or without GnRH agonist pretreatment: A retrospective cohort study stratified by times of embryo implantation failures. Front Endocrinol (Lausanne). 2022;13:803471. 10.3389/fendo.2022.803471 PMC8850772 35185793

[41] Hu X, Yan E, Peng W, Zhou Y, Jin L, Qian K. Higher pre-pregnancy body mass index was associated with adverse pregnancy and perinatal outcomes in women with polycystic ovary syndrome after a freeze-all strategy: a historical cohort study. Acta Obstet Gynecol Scand. 2024;103(5):884–96. 38217337 10.1111/aogs.14771 PMC11019514

[42] Zhou R, Zhang X, Huang L, Wang S, Li L, Dong M, et al. The impact of different cycle regimens on birthweight of singletons in frozen-thawed embryo transfer cycles of ovulatory women. Fertil Steril. 2022;117(3):573–82. 35120746 10.1016/j.fertnstert.2021.09.033

[43] Roelens C, Racca A, Mackens S, Van Landuyt L, Buelinckx L, Gucciardo L, et al. Artificially prepared vitrified-warmed embryo transfer cycles are associated with an increased risk of pre-eclampsia. Reprod Biomed Online. 2022;44(5):915–22. 35282993 10.1016/j.rbmo.2021.12.004

[44] Rodríguez-Eguren A, Bueno-Fernandez C, Gómez-Álvarez M, Francés-Herrero E, Pellicer A, Bellver J, et al. Evolution of biotechnological advances and regenerative therapies for endometrial disorders: a systematic review. Hum Reprod Update. 2024;30(5):584–613. 38796750 10.1093/humupd/dmae013 PMC11369227

[45] Shang Y, Wu M, He R, Ye Y, Sun X. Administration of growth hormone improves endometrial function in women undergoing in vitro fertilization: a systematic review and meta-analysis. Hum Reprod Update. 2022;28(6):838–57. 35641113 10.1093/humupd/dmac028

[46] Yuan G, Yu C, Du X, Li D, Dou H, Lu P, et al. Injectable GelMA hydrogel microspheres with sustained release of Platelet-Rich plasma for the treatment of thin endometrium. Small. 2024;20(47):e2403890. 39206600 10.1002/smll.202403890

[47] Hu KL, Zhang D, Li R. Endometrium preparation and perinatal outcomes in women undergoing single-blastocyst transfer in frozen cycles. Fertil Steril. 2021;115(6):1487–94. 33487443 10.1016/j.fertnstert.2020.12.016

[48] Gu F, Wu Y, Tan M, Hu R, Chen Y, Li X, et al. Programmed frozen embryo transfer cycle increased risk of hypertensive disorders of pregnancy: a multicenter cohort study in ovulatory women. Am J Obstet Gynecol MFM. 2023;5(1):100752. 36115572 10.1016/j.ajogmf.2022.100752

[49] Ginström Ernstad E, Wennerholm UB, Khatibi A, Petzold M, Bergh C. Neonatal and maternal outcome after frozen embryo transfer: increased risks in programmed cycles. Am J Obstet Gynecol. 2019;221(2):126.e1-e18. 10.1016/j.ajog.2019.03.010 30910545

[50] Pereira MM, Mainigi M, Strauss JF. Secretory products of the corpus luteum and preeclampsia. Hum Reprod Update. 2021;27(4):651–72. 33748839 10.1093/humupd/dmab003 PMC8222764

[51] von Versen-Höynck F, Narasimhan P, Selamet Tierney ES, Martinez N, Conrad KP, Baker VL, Winn VD. Absent or excessive corpus luteum number is associated with altered maternal vascular health in early pregnancy. Hypertension. 2019;73(3):680–90. 30636549 10.1161/HYPERTENSIONAHA.118.12046 PMC6378337

[52] von Versen-Höynck F, Schaub AM, Chi YY, Chiu KH, Liu J, Lingis M, et al. Increased preeclampsia risk and reduced aortic compliance with in vitro fertilization cycles in the absence of a corpus luteum. Hypertension. 2019;73(3):640–9. 30636552 10.1161/HYPERTENSIONAHA.118.12043 PMC6434532

[53] Matorras R, Pijoan JI, Laínz L, Díaz-Nuñez M, Sainz H, Pérez-Fernandez S, Moreira D. Polycystic ovarian syndrome and miscarriage in IVF: systematic revision of the literature and meta-analysis. Arch Gynecol Obstet. 2023;308(2):363–77. 36058943 10.1007/s00404-022-06757-0

[54] Vinsonneau L, Labrosse J, Porcu-Buisson G, Chevalier N, Galey J, Ahdad N, et al. Impact of endometrial preparation on early pregnancy loss and live birth rate after frozen embryo transfer: a large multicenter cohort study (14 421 frozen cycles). Hum Reprod Open. 2022;2022(2):hoac007. 35274060 10.1093/hropen/hoac007 PMC8902977

[55] Hatoum I, Bellon L, Swierkowski N, Ouazana M, Bouba S, Fathallah K, et al. Disparities in reproductive outcomes according to the endometrial preparation protocol in frozen embryo transfer: the risk of early pregnancy loss in frozen embryo transfer cycles. J Assist Reprod Genet. 2018;35(3):425–9. 29110260 10.1007/s10815-017-1078-0 PMC5904055

[56] Tomás C, Alsbjerg B, Martikainen H, Humaidan P. Pregnancy loss after frozen-embryo transfer–a comparison of three protocols. Fertil Steril. 2012;98(5):1165–9. 22840239 10.1016/j.fertnstert.2012.07.1058

[57] Xu B, Geerts D, Hu S, Yue J, Li Z, Zhu G, Jin L. The depot GnRH agonist protocol improves the live birth rate per fresh embryo transfer cycle, but not the cumulative live birth rate in normal responders: a randomized controlled trial and molecular mechanism study. Hum Reprod. 2020;35(6):1306–18. 32478400 10.1093/humrep/deaa086

[58] Guo S, Li Z, Yan L, Sun Y, Feng Y. GnRH agonist improves pregnancy outcome in mice with induced adenomyosis by restoring endometrial receptivity. Drug Des Devel Ther. 2018;12:1621–31. 10.2147/DDDT.S162541 PMC5995291 29922037

[59] Ruan HC, Zhu XM, Luo Q, Liu AX, Qian YL, Zhou CY, et al. Ovarian stimulation with GnRH agonist, but not GnRH antagonist, partially restores the expression of endometrial integrin beta3 and leukaemia-inhibitory factor and improves uterine receptivity in mice. Hum Reprod. 2006;21(10):2521–9. 16790614 10.1093/humrep/del215

[60] Song J, Duan C, Cai W, Wu W, Lv H, Xu J. Comparison of GnRH-a prolonged protocol and short GnRH-a long protocol in patients with thin endometrium for assisted reproduction: A retrospective cohort study. Drug Des Devel Ther. 2020;14:3673–82. 10.2147/DDDT.S270519 PMC7505707 32982174

[61] Liu Y, Ma L, Zhu M, Yin H, Yan H, Shi M. Strobe-GnRHa pretreatment in frozen-embryo transfer cycles improves clinical outcomes for patients with persistent thin endometrium: a case-control study. Medicine. 2022;101(31):e29928. 35945767 10.1097/MD.0000000000029928 PMC9351881

