# Supplementary material for: Pregnancy and obstetric-neonatal outcomes of patients with thin endometrium using three different endometrial preparation protocols in frozen embryo transfer cycles: a historical cohort of 2671 patients
Source: Reprod Health. 2025 Oct 15;22:199. doi: 10.1186/s12978-025-02166-z (PMC12522972; doi:10.1186/s12978-025-02166-z)
Supplement: Supplementary file 1 — Supplementary Material 1. Supplemental Table 1. General characteristics and pregnancy outcomes of patients undergoing different endometrial preparation protocols (endometrial thickness < 7 mm). Supplemental Table 2. Crude and adjusted odds ratios of pregnancy outcomes (endometrial thickness < 7 mm). Supplemental Table 3. General characteristics and obstetric-neonatal outcomes of patients with singleton live births (endometrial thickness < 7 mm). Supplemental Table 4. Crude and adjusted odds ratios of obstetric-neonatal outcomes (endometrial thickness < 7 mm). Supplemental Table 5. General characteristics of patients undergoing true natural cycles and modified natural cycles (endometrial thickness < 8 mm). Supplemental Table 6. Crude and adjusted odds ratios of pregnancy and obstetric-neonatal outcomes in true natural cycles and modified natural cycles (endometrial thickness < 8 mm) [file 12978_2025_2166_MOESM1_ESM.docx]

**Supplemental Table 1 General characteristics and pregnancy outcomes of patients undergoing different endometrial preparation protocols (endometrial thickness < 7 mm)**

| **Variable** | **Endometrial preparation protocol** | | | ***P*** |
| --- | --- | --- | --- | --- |
|  | AC (N=768) | GnRH-a + AC (N=220) | NC (N=116) |  |
| Maternal age at oocyte retrieval, years | 33 (30, 37)^a^ | 34 (31, 38)^b^ | 35 (31, 38)^b^ | 0.014^3*^ |
| BMI, kg/m^2^ | 21.36 (19.94, 23.44) | 21.64 (20.02, 23.53) | 21.16 (19.98, 22.96) | 0.307^3^ |
| Baseline FSH, IU/L | 7.37 (6.19, 8.61) | 7.39 (6.24, 9.12) | 7.45 (6.35, 9.21) | 0.414^3^ |
| AFC | 11 (7, 17)^a^ | 9 (6, 16)^b^ | 8 (5, 13)^b^ | <0.001^3*^ |
| AMH, ng/ml | 2.99 (1.60, 5.81)^a^ | 2.49 (1.28, 4.96)^b^ | 2.15 (1.46, 3.48)^b^ | <0.001^3*^ |
| Duration of infertility, years | 2 (1, 4) | 2 (1, 4) | 2 (1, 4) | 0.500^3^ |
| Infertility type |  |  |  | 0.598^1^ |
| Primary infertility | 336 (43.8%) | 96 (43.6%) | 45 (38.8%) |  |
| Second infertility | 432 (56.3%) | 124 (56.4%) | 71 (61.2%) |  |
| Cause of infertility |  |  |  | 0.010^2*^ |
| Tubal factor | 274 (35.7%)^a^ | 88 (40.0%)^a^ | 43 (37.1%)^a^ |  |
| Male factor | 76 (9.9%)^a^ | 14 (6.4%)^a^ | 10 (8.6%)^a^ |  |
| Diminished ovarian reserve | 95 (12.4%)^a^ | 41 (18.6%)^a^ | 15 (12.9%)^a^ |  |
| Ovulatory disorders | 59 (7.7%)^a^ | 9 (4.1%)^a^ | 0 (0.0%)^b^ |  |
| Endometriosis | 15 (2.0%)^a^ | 10 (4.5%)^a^ | 1 (0.9%)^a^ |  |
| Unexplained | 27 (3.5%)^a,b^ | 4 (1.8%)^b^ | 6 (5.2%)^a^ |  |
| Uterine | 71 (9.2%)^a^ | 11 (5.0%)^a^ | 13 (11.2%)^a^ |  |
| Mixed | 151 (19.7%)^a^ | 43 (19.5%)^a^ | 28 (24.1%)^a^ |  |
| COH protocol |  |  |  | 0.004^1*^ |
| Depot GnRH-a | 97 (12.6%)^a^ | 14 (6.4%)^b^ | 7 (6.0%)^a,b^ |  |
| Long GnRH-a | 238 (31.0%)^a^ | 57 (25.9%)^a^ | 27 (23.3%)^a^ |  |
| GnRH antagonist | 316 (41.1%)^a^ | 102 (46.4%)^a^ | 59 (50.9%)^a^ |  |
| Other protocols | 117 (15.2%)^a^ | 47 (21.4%)^a^ | 23 (19.8%)^a^ |  |
| Fertilisation method |  |  |  | 0.334^1^ |
| IVF | 587 (76.4%) | 168 (76.4%) | 94 (81.0%) |  |
| ICSI | 156 (20.3%) | 41 (18.6%) | 16 (13.8%) |  |
| IVF+RICSI | 25 (3.3%) | 11 (5.0%) | 6 (5.2%) |  |
| Gonadotrophin duration, days | 10 (8, 11) | 9 (9, 11) | 9 (8, 10) | 0.171^3^ |
| Gonadotrophin dose, IU | 2400.0 (1800.0, 3000.0) | 2475.0 (1918.0, 3150.0) | 2475.0 (1950.0, 3018.5) | 0.160^3^ |
| No. of oocytes retrieved | 11 (7, 17)^a^ | 10 (6, 15)^b^ | 11 (6, 14)^b^ | 0.038^3*^ |
| No. of MⅡ oocytes | 10 (6, 14) | 9 (5, 13) | 9 (5, 13) | 0.081^3^ |
| No. of 2PN | 7 (4, 10) | 6 (4, 9) | 7 (4, 10) | 0.386^3^ |
| Normal fertilisation rate | 0.73 (0.60, 0.85) | 0.72 (0.60, 0.86) | 0.77 (0.67, 0.91) | 0.051^3^ |
| Blastocyst formation rate | 0.67 (0.33, 0.87) | 0.64 (0.33, 0.86) | 0.67 (0.30, 0.83) | 0.538^3^ |
| Maternal age at FET, years | 34 (31, 38)^a^ | 35 (32, 39)^b^ | 36 (32, 38)^b^ | 0.002^3*^ |
| Endometrial thickness, mm | 6.2 (5.8, 6.6) | 6.1 (5.4, 6.6) | 6.2 (5.7, 6.6) | 0.052^3^ |
| No. of embryos transferred |  |  |  | 0.556^1^ |
| One | 578 (75.3%) | 158 (71.8%) | 88 (75.9%) |  |
| Two | 190 (24.7%) | 62 (28.2%) | 28 (24.1%) |  |
| Type of embryo transferred |  |  |  | 0.541^1^ |
| Day 3 (cleavage stage) | 215 (28.0%) | 70 (31.8%) | 33 (28.4%) |  |
| Day 5/6 (blastocyst stage) | 553 (72.0%) | 150 (68.2%) | 83 (71.6%) |  |
|  |  |  |  |  |
| **Pregnancy outcomes** |  |  |  |  |
| Biochemical pregnancy loss | 62 (8.1%) | 14 (6.4%) | 3 (2.6%) | 0.090^2^ |
| Clinical pregnancy | 213 (27.7%) | 57 (25.9%) | 35 (30.2%) | 0.703^1^ |
| Live birth | 148 (19.3%) | 37 (16.8%) | 27 (23.3%) | 0.359^1^ |
| Multiple pregnancies | 21 (2.7%) | 5 (2.3%) | 6 (5.2%) | 0.305^2^ |
| Ectopic pregnancy | 5 (0.7%) | 1 (0.5%) | 0 (0.0%) | 0.852^2^ |
| Miscarriage | 63 (29.6%) | 20 (35.1%) | 8 (22.9%) | 0.456^1^ |

Note: AC = artificial cycle; GnRH-a = gonadotropin-releasing hormone agonist; NC = natural cycle; BMI = body mass index; FSH = follicle stimulation hormone; AFC = antral follicle count; AMH = anti-müllierian hormone; COH = controlled ovarian hyperstimulation; IVF = *in-vitro* fertilisation; ICSI = intracytoplasmic sperm injection; RICSI = rescue ICSI; MII = metaphase II; PN = Pronucleus; FET = frozen embryo transfer.

^1^ Chi-Square p-value; ^2^ Fisher Exact p-value; ^3^ Kruskal-Wallis p-value. ^a, b^**Statistically significant differences between groups after Bonferroni correction. Groups sharing the same letter are not significantly different.** * *P* < 0.05 (without **Bonferroni correction**).

Data are median (interquartile range) or n (%) unless otherwise specified.

**Supplemental Table 2 Crude and adjusted odds ratios of pregnancy outcomes (endometrial thickness < 7 mm)**

| **Variable** | **AC vs. NC** | | **AC vs. GnRH-a + AC** | | **NC vs. GnRH-a + AC** | |
| --- | --- | --- | --- | --- | --- | --- |
|  | Crude OR (95%CI) | Adjusted OR (95%CI) | Crude OR (95%CI) | Adjusted OR (95%CI) | Crude OR (95%CI) | Adjusted OR (95%CI) |
| Biochemical pregnancy loss ^a^ | **0.302**  **(0.093, 0.979)** | 0.333  (0.102, 1.093) | 0.774  (0.425, 1.411) | 0.742  (0.397, 1.385) | 2.560  (0.720, 9.096) | 2.226  (0.615, 8.049) |
| Clinical pregnancy ^b^ | 1.126  (0.735, 1.725) | 1.310  (0.835, 2.054) | 0.911  (0.648, 1.281) | 1.053  (0.731, 1.516) | 0.809  (0.492, 1.332) | 0.804  (0.476, 1.358) |
| Live birth ^b^ | 1.271  (0.797, 2.026) | 1.480  (0.908, 2.415) | 0.847  (0.570, 1.259) | 1.016  (0.669, 1.543) | 0.666  (0.382, 1.163) | 0.686  (0.384, 1.226) |
| Multiple pregnancy ^c^ | 1.995  (0.787, 5.054) | 2.653  (0.951, 7.401) | 0.827  (0.308, 2.220) | 0.944  (0.328, 2.715) | 0.415  (0.124, 1.390) | 0.356  (0.098, 1.297) |
| Ectopic pregnancy ^d^ | NA | NA | 0.697 (0.081, 5.996) | 0.584 (0.064, 5.377) | NA | NA |
| Miscarriage ^e^ | 0.835  (0.380, 1.835) | 0.478  (0.198, 1.153) | 1.217  (0.662, 2.240) | 1.065  (0.531, 2.137) | 1.458  (0.587, 3.622) | 2.226  (0.805, 6.153) |
|  | Referred to AC | | Referred to AC | | Referred to NC | |

Note: Odds ratios (ORs) and 95% confidence intervals (CIs) are based on the univariate analysis. Adjusted odds ratios (AORs) and 95% CIs are based on the multiple logistic regression model.

^a^ Adjusted for maternal age at FET, BMI, cause of infertility, number of retrieved oocytes, infertility type, duration of infertility, controlled ovarian hyperstimulation (COH) protocol, type and number of embryos transferred.

^b^ Adjusted for maternal age at FET, BMI, cause of infertility, fertilisation method, number of retrieved oocytes, type and number of embryos transferred, and endometrial thickness.

^c^ Adjusted for maternal age at FET, BMI, cause of infertility, number of retrieved oocytes, COH protocol, type and number of embryos transferred.

^d^ Adjusted for maternal age at FET, BMI, cause of infertility, and number of retrieved oocytes.

^e^ Adjusted for maternal age at FET, BMI, cause of infertility, fertilisation method, number of retrieved oocytes, COH protocol, number of embryos transferred, and endometrial thickness.

NA: Insufficient data for statistical analysis.

AC = artificial cycle; NC = natural cycle; GnRH-a = gonadotropin-releasing hormone agonist; OR = odds ratios; CI = confidence interval; FET = frozen embryo transfer; BMI = body mass index; COH = controlled ovarian hyperstimulation.

**Supplemental Table 3 General characteristics and obstetric-neonatal outcomes of patients with singleton live births (endometrial thickness < 7 mm)**

| **Variable** | **Endometrial preparation protocol** | | | ***P*** |
| --- | --- | --- | --- | --- |
|  | AC (N=141) | GnRH-a + AC (N=34) | NC (N=25) |  |
| Maternal age at oocyte retrieval, years | 31 (21, 35) | 32 (30, 37) | 34 (30, 37) | 0.095^3^ |
| BMI, kg/m^2^ | 21.52 (19.82, 23.72) | 21.43 (20.02, 23.75) | 21.22 (20.31, 25.61) | 0.862^3^ |
| Baseline FSH, IU/L | 7.35 (6.15, 8.46) | 7.08 (6.00, 8.04) | 7.35 (5.55, 8.39) | 0.705^3^ |
| AFC | 12 (7, 19) | 10 (6, 18) | 9 (6, 15) | 0.180^3^ |
| AMH, ng/ml | 3.30 (1.45, 6.34) | 3.39 (1.91, 7.20) | 2.18 (1.40, 3.64) | 0.087^3^ |
| Duration of infertility, years | 2 (1, 5) | 2 (1, 3) | 2 (1, 4) | 0.261^3^ |
| Infertility type |  |  |  | 0.357^1^ |
| Primary infertility | 67 (47.5%) | 12 (35.3%) | 13 (52.0%) |  |
| Second infertility | 74 (52.5%) | 22 (64.7%) | 12 (48.0%) |  |
| Cause of infertility |  |  |  | 0.048^2*^ |
| Tubal factor | 45 (31.9%)^a^ | 13 (38.2%)^a^ | 12 (48.0%)^a^ |  |
| Male factor | 17 (12.1%)^a^ | 2 (5.9%)^a^ | 3 (12.0%)^a^ |  |
| Diminished ovarian reserve | 8 (5.7%)^a^ | 4 (11.8%)^a,b^ | 6 (24.0%)^b^ |  |
| Ovulatory disorders | 17 (12.1%)^a^ | 3 (8.8%)^a^ | 0 (0.0%)^a^ |  |
| Endometriosis | 1 (0.7%)^a^ | 2 (5.9%)^a^ | 0 (0.0%)^a^ |  |
| Unexplained | 8 (5.7%)^a^ | 2 (5.9%)^a^ | 1 (4.0%)^a^ |  |
| Uterine | 10 (7.1%)^a^ | 3 (8.8%)^a^ | 2 (8.0%)^a^ |  |
| Mixed | 35 (24.8%)^a^ | 5 (14.7%)^a^ | 1 (4.0%)^a^ |  |
| COH protocol |  |  |  | 0.025^2*^ |
| Depot GnRH-a | 14 (9.9%)^a^ | 1 (2.9%)^a^ | 2 (8.0%)^a^ |  |
| Long GnRH-a | 49 (34.8%)^a^ | 9 (26.5%)^a^ | 4 (16.0%)^a^ |  |
| GnRH antagonist | 54 (38.3%)^a^ | 22 (64.7%)^b^ | 17 (68.0%)^b^ |  |
| Other protocols | 24 (17.0%)^a^ | 2 (5.9%)^a^ | 2 (8.0%)^a^ |  |
| Fertilisation method |  |  |  | 0.245^1^ |
| IVF | 108 (76.6%) | 27 (79.4%) | 19 (76.0%) |  |
| ICSI | 33 (23.4%) | 6 (17.7%) | 5 (20.0%) |  |
| IVF+RICSI | 0 (0.0%) | 1 (2.9%) | 1 (4.0%) |  |
| No. of oocytes retrieved | 12 (7, 19) | 12 (8, 18) | 11 (7, 17) | 0.750^3^ |
| No. of MⅡ oocytes | 10 (7, 17) | 11 (7, 15) | 6 (10, 15) | 0.835^3^ |
| No. of 2PN | 7 (4, 12) | 8 (5, 11) | 5 (7, 11) | 0.954^3^ |
| Normal fertilisation rate | 0.74 (0.63, 0.83) | 0.73 (0.66, 0.84) | 0.75 (0.65, 0.88) | 0.558^3^ |
| Blastocyst formation rate | 0.68 (0.38, 0.84) | 0.73 (0.33, 0.88) | 0.67 (0.50, 0.90) | 0.918^3^ |
| Maternal age at FET, years | 32 (29, 35)^a^ | 32 (31, 35)^a,b^ | 35 (31, 38)^b^ | 0.015^3*^ |
| Endometrial thickness, mm | 6.4 (6.0, 6.7) | 6.6 (6.0, 6.8) | 6.5 (5.5, 6.8) | 0.420^3^ |
| No. of embryos transferred |  |  |  | 0.817^2^ |
| One | 115 (81.6%) | 27 (79.4%) | 19 (76.0%) |  |
| Two | 26 (18.4%) | 7 (20.6%) | 6 (24.0%) |  |
| Type of embryo transferred |  |  |  | 0.586^2^ |
| Day 3 (cleavage stage) | 23 (16.3%) | 6 (17.6%) | 2 (8.0%) |  |
| Day 5/6 (blastocyst stage) | 118 (83.7%) | 28 (82.4%) | 23 (92.0%) |  |
|  |  |  |  |  |
| **Obstetric-neonatal outcomes** |  |  |  |  |
| Delivery mode |  |  |  | 0.451^2^ |
| Caesarean delivery | 128 (90.8%) | 32 (94.1%) | 21 (84.0%) |  |
| Natural labor | 13 (9.2%) | 2 (5.9%) | 4 (16.0%) |  |
| Gender |  |  |  | 0.576^1^ |
| Male | 78 (55.3%) | 16 (47.1%) | 15 (60.0%) |  |
| Female | 63 (44.7%) | 18 (52.9%) | 10 (40.0%) |  |
| Gestational age, weeks | 38.57 (37.43, 39.14) | 38.29 (37.14, 38.86) | 38.29 (37.00, 38.86) | 0.325^3^ |
| Birth weight, kg | 3.20 (2.90, 3.50) | 3.10 (2.92, 3.42) | 3.15 (2.93, 3.30) | 0.576^3^ |
| Low birth weight < 2, 500 g | 14 (9.9%) | 4 (11.8%) | 2 (8.0%) | 0.870^2^ |
| Macrosomia > 4, 000 g | 7 (5.0%) | 0 (0.0%) | 0 (0.0%) | 0.222^2^ |
| Small for gestational age | 5 (3.5%) | 2 (5.9%) | 0 (0.0%) | 0.596^2^ |
| Large for gestational age | 20 (14.2%) | 2 (5.9%) | 0 (0.0%) | 0.052^2^ |
| Preterm birth | 22 (15.6%) | 7 (20.6%) | 6 (24.0%) | 0.536^2^ |
| Gestational diabetes mellitus | 3 (2.1%) | 1 (2.9%) | 0 (0.0%) | >0.999^2^ |
| Hypertensive disorders of pregnancy | 7 (5.0%) | 2 (5.9%) | 2 (8.0%) | 0.886^2^ |
| Placenta previa | 3 (2.1%) | 1 (2.9%) | 1 (4.0%) | >0.999^2^ |
| Fetal malformation | 2 (1.4%) | 0 (0.0%) | 0 (0.0%) | >0.999^2^ |

Note: ^1^ Chi-Square p-value; ^2^ Fisher Exact p-value; ^3^ Kruskal-Wallis p-value. ^a, b^**Statistically significant differences between groups after Bonferroni correction. Groups sharing the same letter are not significantly different.** * *P* < 0.05 (without **Bonferroni correction**).

Data are median (interquartile range) or n (%) unless otherwise specified.

AC = artificial cycle; GnRH-a = gonadotropin-releasing hormone agonist; NC = natural cycle; BMI = body mass index; FSH = follicle stimulation hormone; AFC = antral follicle count; AMH = anti-müllierian hormone; COH = controlled ovarian hyperstimulation; IVF = *in-vitro* fertilization; ICSI = intracytoplasmic sperm injection; RICSI = rescue ICSI; MII = metaphase II; PN = Pronucleus; FET = frozen embryo transfer.

**Supplemental Table 4 Crude and adjusted odds ratios of obstetric-neonatal outcomes (endometrial thickness < 7 mm)**

| **Variable** | **AC vs. NC** | | **AC vs. GnRH-a + AC** | | **NC vs. GnRH-a + AC** | |
| --- | --- | --- | --- | --- | --- | --- |
|  | Crude OR  (95% CI) | Adjusted OR (95% CI) | Crude OR  (95% CI) | Adjusted OR (95% CI) | Crude OR  (95% CI) | Adjusted OR (95% CI) |
| Low birth weight < 2, 500g ^a^ | 0.789  (0.168, 3.704) | 0.996  (0.181, 5.477) | 1.210  (0.372, 3.937) | 1.494  (0.386, 5.788) | 1.533  (0.258, 9.112) | 1.501  (0.222, 10.147) |
| Small for gestational age ^b^ | NA | NA | 1.700  (0.315, 9.162) | 1.067  (0.165, 6.909) | NA | NA |
| Large for gestational age ^b^ | NA | NA | 0.378  (0.084, 1.703) | 0.367  (0.079, 1.703) | NA | NA |
| Preterm birth ^a^ | 1.694  (0.608, 4.718) | 2.064  (0.675, 6.312) | 1.391  (0.539, 3.588) | 1.486  (0.541, 4.080) | 0.821  (0.238, 2.832) | 0.720  (0.196, 2.641) |
| Gestational diabetes mellitus ^c^ | NA | NA | 1.384  (0.139, 13.732) | 1.023  (0.069, 15.144) | NA | NA |
| Hypertensive disorders of pregnancy ^b^ | 1.652  (0.323, 8.455) | 1.406  (0.235, 8.411) | 1.187  (0.235, 5.990) | 1.142  (0.213, 6.139) | 0.719  (0.094, 5.483) | 0.813  (0.100, 6.637) |
| Placenta previa ^d^ | 1.903  (0.190, 19.061) | 2.659  (0.232, 30.532) | 1.384  (0.139, 13.732) | 3.126  (0.178, 54.962) | 0.727  (0.043, 12.216) | 0.352  (0.010, 11.852) |
|  | Referred to AC | | Referred to AC | | Referred to NC | |

Odds ratios (ORs) and 95% confidence intervals (CIs) are based on the univariate analysis. Adjusted odds ratios (AORs) and 95% CIs are based on the multiple logistic regression model.

^a^ Adjusted for maternal age at FET, BMI, cause of infertility, and duration of infertility.

^b^ Adjusted for maternal age at FET, BMI and cause of infertility.

^c^ Adjusted for maternal age at FET, BMI, cause of infertility and number of retrieved oocytes.

NA: Insufficient data for statistical analysis.

AC = artificial cycle; NC = natural cycle; GnRH-a = gonadotropin-releasing hormone agonist; OR = odds ratios; CI = confidence interval; FET = frozen embryo transfer; BMI = body mass index.

**Supplemental Table 5 General characteristics of patients undergoing true natural cycles and modified natural cycles (endometrial thickness < 8 mm)**

| **Variable** | **Endometrial preparation protocol** | | ***P*** |
| --- | --- | --- | --- |
|  | Modified NC (N=211) | True NC (N=46) |  |
| Maternal age at oocyte retrieval, years | 34 (31, 38) | 33 (29, 39) | 0.866^3^ |
| BMI, kg/m2 | 21.03 (19.72, 22.94) | 22.00 (20.81, 24.24) | 0.043^3*^ |
| Baseline FSH, IU/L | 7.61 (6.35, 9.22) | 7.41 (6.22, 8.53) | 0.303^3^ |
| AFC | 9 (6, 13) | 10 (5, 14) | 0.686^3^ |
| AMH, ng/ml | 2.15 (1.25, 3.73) | 2.86 (1.52, 4.78) | 0.244^3^ |
| Duration of infertility, years | 2 (1, 4) | 3 (2, 4) | 0.130^3^ |
| Infertility type |  |  | 0.466^1^ |
| Primary infertility | 95 (45.0%) | 18 (39.1%) |  |
| Second infertility | 116 (55.0%) | 28 (60.9%) |  |
| Cause of infertility |  |  | 0.981^2^ |
| Tubal factor | 66 (31.3%) | 17 (37.0%) |  |
| Male factor | 34 (16.1%) | 6 (13.0%) |  |
| Diminished ovarian reserve | 34 (16.1%) | 8 (17.4%) |  |
| Endometriosis | 3 (1.4%) | 0 (0.0%) |  |
| Unexplained | 14 (6.6%) | 3 (6.5%) |  |
| Uterine | 19 (9.0%) | 4 (8.7%) |  |
| Mixed | 41 (19.4%) | 8 (17.4%) |  |
| COH protocol |  |  | 0.708^1^ |
| Depot GnRH-a | 18 (8.5%) | 6 (13.0%) |  |
| Long GnRH-a | 51 (24.2%) | 9 (19.6%) |  |
| GnRH antagonist | 100 (47.4%) | 21 (45.7%) |  |
| Other protocols | 42 (19.9%) | 10 (21.7%) |  |
| Fertilisation method |  |  | 0.311^2^ |
| IVF | 158 (74.9%) | 30 (65.2%) |  |
| ICSI | 42 (19.9%) | 14 (30.4%) |  |
| IVF+RICSI | 11 (5.2%) | 2 (4.3%) |  |
| Gonadotrophin duration, days | 9 (9, 11) | 9 (8, 11) | 0.120^3^ |
| Gonadotrophin dose, IU | 2550.0 (2025.0, 3075.0) | 2325.0 (1800.0, 3000.0) | 0.401^3^ |
| No. of oocytes retrieved | 10 (5, 14) | 11 (7, 18) | 0.430^3^ |
| No. of MⅡ oocytes | 9 (5, 12) | 9 (6, 15) | 0.766^3^ |
| No. of 2PN | 6 (4, 10) | 6 (3, 10) | 0.765^3^ |
| Normal fertilisation rate | 0.75 (0.63,0.90) | 0.75 (0.50, 0.88) | 0.449^3^ |
| Blastocyst formation rate | 0.67 (0.20, 0.88) | 0.67 (0.33, 0.80) | 0.641^3^ |
| Maternal age at FET, years | 35 (32, 39) | 34 (31, 39) | 0.953^3^ |
| Endometrial thickness, mm | 7.0 (6.2, 7.5) | 7.2 (6.4, 7.6) | 0.378^3^ |
| No. of embryos transferred |  |  | 0.264^1^ |
| One | 158 (74.9%) | 38 (82.6%) |  |
| Two | 53 (25.1%) | 8 (17.4%) |  |
| Type of embryo transferred |  |  | 0.762^1^ |
| Day 3 (cleavage stage) | 64 (30.3%) | 15 (32.6%) |  |
| Day 5/6 (blastocyst stage) | 147 (69.7%) | 31 (67.4%) |  |

Note: NC = natural cycle; BMI = body mass index; FSH = follicle stimulation hormone; AFC = antral follicle count; AMH = anti-müllierian hormone; COH = controlled ovarian hyperstimulation; IVF = in-vitro fertilisation; ICSI = intracytoplasmic sperm injection; RICSI = rescue ICSI; MII = metaphase II; PN = Pronucleus; FET = frozen embryo transfer.

1 Chi-Square p-value; 2 Fisher Exact p-value; 3 Kruskal-Wallis p-value; * *P* < 0.05

Data are median (interquartile range) or n (%) unless otherwise specified.

**Supplemental Table 6 Crude and adjusted odds ratios of pregnancy and obstetric-neonatal outcomes in true natural cycles and modified natural cycles (endometrial thickness < 8 mm)**

| **Variable** | **Modified NC** | **True NC** | ***P*** | **Crude OR (95%CI)** | **Adjusted OR (95%CI)** |
| --- | --- | --- | --- | --- | --- |
| **Pregnancy outcomes** |  |  |  |  |  |
| Biochemical pregnancy loss | 10/211 (4.7%) | 0/46 (0.0%) | 0.122 | NA | NA |
| Clinical pregnancy ^a^ | 72/211 (34.1%) | 15/46 (32.6%) | 0.844 | 1.071 (0.543, 2.111) | 1.271 (0.585, 2.762) |
| Live birth ^a^ | 60/211 (28.4%) | 11/46 (23.9%) | 0.534 | 1.264 (0.603, 2.651) | 1.503 (0.629, 3.591) |
| Ectopic pregnancy | 0/211 (0.0%) | 0/46 (0.0%) | NA | NA | NA |
| Miscarriage ^b^ | 12/72 (16.7%) | 4/15 (26.7%) | 0.282 | 0.550 (0.150, 2.021) | 0.479 (0.064, 3.616) |
|  |  |  |  |  |  |
| **Obstetric-neonatal outcomes** |  |  |  |  |  |
| Low birth weight < 2, 500 g ^c^ | 3/51 (5.9%) | 1/11 (9.1%) | 0.552 | 0.625 (0.059, 6.643) | 0.520 (0.044, 6.112) |
| Macrosomia > 4, 000 g | 1/51 (2.0%) | 0/11 (0.0%) | 0.823 | NA | NA |
| Small for gestational age | 1/51 (2.0%) | 0/11 (0.0%) | 0.823 | NA | NA |
| Large for gestational age | 6/51 (11.8%) | 0/11 (0.0%) | 0.293 | NA | NA |
| Preterm birth ^d^ | 9/51 (17.6%) | 1/11 (9.1%) | 0.430 | 2.143 (0.243, 18.919) | 3.288 (0.197, 54.994) |
| Gestational diabetes mellitus | 1/51 (2.0%) | 0/11 (0.0%) | 0.823 | NA | NA |
| HDP | 5/51 (9.8%) | 0/11 (0.0%) | 0.363 | NA | NA |
| Placenta previa | 0/51 (0.0%) | 0/11 (0.0%) | NA | NA | NA |
| Fetal malformation | 0/51 (0.0%) | 0/11 (0.0%) | NA | NA | NA |
|  |  |  |  | Referred to true NC | |

Note: Odds ratios (ORs) and 95% confidence intervals (CIs) are based on the univariate analysis. Adjusted odds ratios (AORs) and 95% CIs are based on the multiple logistic regression model.

a Adjusted for maternal age at FET, BMI, cause of infertility, number of retrieved oocytes, infertility type, duration of infertility, COH protocol, type and number of embryos transferred.

b Adjusted for maternal age at FET, BMI, cause of infertility, fertilisation method, number of retrieved oocytes, COH protocol, number of embryos transferred, and endometrial thickness.

c Adjusted for maternal age at FET, BMI, cause of infertility, duration of infertility, and number of retrieved oocytes.

d Adjusted for maternal age at FET, BMI, cause of infertility, infertility type, and number of retrieved oocytes.

NA: Insufficient data for statistical analysis.

NC = natural cycle; OR = odds ratios; CI = confidence interval; HDP = hypertensive disorders of pregnancy; FET = frozen embryo transfer; BMI = body mass index; COH = controlled ovarian hyperstimulation.
